# Efficacy and Safety of Xingnaojing Injection for Emergency Treatment of Acute Ischemic Stroke: A Systematic Review and Meta-Analysis

**DOI:** 10.3389/fphar.2022.839305

**Published:** 2022-03-24

**Authors:** Liuding Wang, Xueming Fan, Yifan Chen, Xiao Liang, Wei Shen, Yunling Zhang

**Affiliations:** Xiyuan Hospital, China Academy of Chinese Medical Sciences, Beijing, China

**Keywords:** Xingnaojing injection, acute ischemic stroke, efficacy, safety, systematic review, meta-analysis

## Abstract

**Background:** Xingnaojing injection (XNJ) is derived from a traditional Chinese prescription named Angong Niuhuang pill. As an adjuvant treatment widely used in acute ischemic stroke (AIS), XNJ has proven to be effective with certain clinical evidence. The aim of this study is to collect the latest evidence and evaluate efficacy and safety of XNJ for emergency treatment of AIS.

**Methods:** We searched seven literature databases and two clinical trial registries from their inception to November 14, 2021 for randomized controlled trials (RCTs) examining the efficacy of XNJ for AIS. Two reviewers independently selected relevant trials, extracted data, and assessed the risk of bias. We pooled data into a meta-analysis using RevMan 5.4 software.

**Results:** Thirty-eight RCTs were included in this review, with a total of 3,677 participants. XNJ plus conventional treatments (CTs) showed a significant advantage, compared with CTs alone, in improving functional independence at 14 days (*RR* = 1.70, 95% *CI* = 1.03 to 2.81, *p* = 0.04), neurological function (*MD*_NIHSS < 6h_ = −3.81, 95% *CI* = −5.25 to −2.38, *p* < 0.00001; *MD*_NIHSS < 24h_ = −3.75, 95% *CI* = −4.92 to −2.59, *p* < 0.00001; *MD*_NIHSS < 72h_ = −3.74, 95% *CI* = −5.48 to −2.00, *p* < 0.0001; *MD*_NIHSS < 14d_ = −1.97, 95% *CI* = −3.25 to −0.69, *p* = 0.003), and activities of daily living on the Barthel index (*MD*_BI-14day_ = 9.97, 95% *CI* = 9.29 to 10.65, *p* < 0.00001; *MD*_BI-30day_ = 10.04, 95% *CI* = 5.82, to 14.26, *p* < 0.00001). In addition, the results showed that XNJ plus CTs was superior to CTs alone in reducing IL-6, TNF-α, hs-CRP, and MMP-9. Regarding safety of XNJ, the incidence of adverse reactions in the XNJ group was lower than that in the control group (*RR* = 0.57, 95% *CI* = 0.38 to 0.87, *p* = 0.009). The certainty of evidence was evaluated as low or very low for all.

**Conclusion:** XNJ appears to be effective and safe for emergency treatment of AIS. The first 72 h after the onset of stroke, in particular the first 6 hours, may be the optimum initiation time. However, further high-quality RCTs are warranted to determine an appropriate initiation time.

**Systematic Review Registration:** [https://www.crd.york.ac.uk/PROSPERO/display_record.php?RecordID=233211], identifier [CRD42021233211].

## Introduction

Acute ischemic stroke (AIS) is a life-threatening medical condition that generally carries poor prognosis due to untimely treatment. There was a relative increase of roughly 8.9% in its global lifetime risk over the past 3 decades (Feigin et al., 2018). Stroke is the prominent cause of mortality and disability worldwide (Campbell and Khatri, 2020), and ischemic stroke accounts for about 80% (Donkor, 2018) of it, among which AIS is particularly dangerous. A Chinese large cohort study showed that the patients with AIS had a high rate of in-hospital recurrence of approximately 5.7% in the first 5 days, resulting in a higher in-hospital mortality rate (Yu et al., 2019). Its rate of death/disability within 3 months of onset in China was as high as 34.5–37.1% (Hao et al., 2011; Wang et al., 2013). To make matters worse, the incidence of stroke continues to increase with one in four people worldwide predicted to suffer from stroke during their lifetime (Collaborators, 2018). Therefore, AIS, bringing about a substantial economic and social burden, is of immense public health impact. Due to the demographic transitions, the public health burden is set to further rise over future decades (Adogu et al., 2015).

At present, the first-line emergency treatments for AIS recommended by the American Heart Association/American Stroke Association (AHA/ASA) are intravenous thrombolysis (IVT) and endovascular therapies (EVTs) (Powers et al., 2019). However, to date, both IVT and EVTs have low implementation rates due to the narrow therapeutic window and hemorrhagic transformation (Tao et al., 2018; Hasnain et al., 2020; Sharobeam et al., 2021). Consequently, no other effective emergency therapies are available to address acute pathological reactions.

Angong Niuhuang pill is a first-aid Chinese patent medicine for acute stroke with more than 200 years of clinical application (Liu C et al., 2019). With the approval of the China Food and Drug Administration, Xingnaojing injection (XNJ), a derivative of Angong Niuhuang pill, has been widely used already as an emergency treatment in the acute stage of cerebral infarction (Liu et al., 2010). XNJ, extracted from Chinese botanical drugs *via* steam distillation, comprises *Dryobalanops aromatica* C.F.Gaertn [Dipterocarpaceae; Borneolum], *Curcuma aromatica* Salisb. [Zingiberaceae; Curcumae Radix], *Gardenia jasminoides* J. Ellis [Rubiaceae; Gardeniae Fructus], *Moschus berezovskii* Flerov, *M. sifanicus* Przewalski, or *M. moschiferus* Linnaeus [Cervidae; Moschus]. As for the process of preparation, 30 g of Curcumae Radix and 30 g of Gardeniae Fructus are first distilled with 1,500 ml of water, from which 1,000 ml of the distillate is collected; 7.5 g of Moschus and 250 ml of distilled water are then added to the abovementioned distillate, and 1,000 ml of the distillate is collected for standby; 1 g of Borneolum and 8 g of polysorbate 80 are ground and added to the distillate; finally, 8 g of sodium chloride is added, and the distillate is stirred, mixed, placed, and refrigerated overnight and then filtered, potted, and sterilized (Pharmacopoeia Committee of the Ministry of Public Health of the People’s Republic of China, 1998). With regard to the identified active components, borneol, whose concentration is traditionally used to control the quality of XNJ, should not be less than 0.7 g/L in accordance with the drug standards of the China Food and Drug Administration (China Food and Drug Administration, 2003). Moreover, by using gas chromatography–mass spectrometry (GC-MS), network pharmacology, and molecular docking technology, researchers recently found that the representative active components of XNJ also include geniposide, curdione, and muscone (Wu et al., 2021). Over the past few years, even though many studies have shown that the abovementioned components could inhibit oxidation, promote anti-inflammation, regulate the apoptosis, and activate autophagy (Liu et al., 2017; Lee et al., 2019; Fu et al., 2020; Zhang C et al., 2020), the complex mechanisms of this multiherbal preparation in cerebral infarction have been under exploration. Notably, in a rat model of middle cerebral artery occlusion–reperfusion, researchers have confirmed *in vivo* efficacy that XNJ could protect nerve cells and improve cerebral ischemia–reperfusion injury and conducted a preliminary investigation of the anti-inflammatory mechanisms, which probably relate to suppressing NLRP3 inflammasomes and enhancing SIRT1 expression (Zhang et al., 2018; Qu et al., 2019; Zhang Y et al., 2020). Furthermore, as for a therapeutically relevant dose range, an experiment has found that rats injected with 10 or 15 ml/kg of XNJ 24 h before ischemia and at the onset of reperfusion, respectively, showed greater improvement in neurological function and infarct volume with statistical significance than those injected with saline injection or 5 ml/kg of XNJ (Zhang et al., 2018). A systematic review of animal studies also supported these preclinical evidences (Ma et al., 2018). In the theory of traditional Chinese medicine (TCM), XNJ has effects of *Qingre Jiedu* (a TCM term means clearing heat and detoxification) and *Huoxue Huayu* (a TCM term means promoting blood circulation and removing blood stasis) (Xu, 2010). Therefore, it can act on the critical pathological factors, heat toxin, and blood stagnation during the acute phase of ischemic stroke.

Continued evaluation of clinical efficacy from meta-analyses indicates that XNJ can benefit patients with ischemic stroke (Peng et al., 2014; Ma et al., 2017; Tian et al., 2021). A previous systematic review (Peng et al., 2014) of thirteen trials published in 2014 suggested that the efficacy and safety of XNJ in stroke patients were inconclusive, and it lacked subgroup analysis according to the types of strokes. The latest systematic review (Ma et al., 2017) of 53 trials published in 2017 concluded that XNJ might be a beneficial therapeutic method for cerebral infarction. However, lack of description regarding time to initiate XNJ has led to unclear efficacy evaluation of XNJ as a first-aid medicine in the acute phase. It was uncertain whether XNJ can be used immediately after the symptom onset. An overview of systematic reviews (Tian et al., 2021) pointed out that previous systematic reviews of XNJ for ischemic stroke had the following problems: 1) some critical items of AMSTAR2 were poorly reported, which included predefined protocol, comprehensive search strategy, list of excluded studies, and reasons for exclusion. 2) Primary outcomes have always been measured using the total effective rate and neurological function instead of the modified Rankin Scale (mRS). 3) The clinical benefit in the acute phase of ischemic stroke is unclear.

To some extent, this review has sought to avoid the abovementioned limitations, providing relatively complete and up-to-date evidence on the use of XNJ for emergency treatment of AIS. The functional independence rate is used as the primary outcome. In addition, reporting of this review is in strict accordance with the Preferred Reporting Items for Systematic Review and Meta-Analyses (PRISMA) 2020 statement (Page et al., 2021). Thus, this quantitative review aimed at answering the following questions: What is the additional clinical benefit of XNJ+conventional treatments (CTs) compared to CTs alone on AIS patients? What is the optimum initiation time of XNJ on AIS patients? To what extent is XNJ safe to be administered on AIS patients?

## Methods

### Protocol Register

The protocol of this systematic review was prospectively registered in PROSPERO (Registration Number: CRD42021233211).

### Search Strategy

We searched the following databases and registries from their inception to January 2021: PubMed, Cochrane Library, Embase, ClinicalTrials.gov, Chinese Biomedical Literature Service System (SinoMed), China National Knowledge Infrastructure (CNKI), Chinese Scientific Journals Database (VIP), WanFang database, and Chinese Clinical Trial Register (ChiCTR). The detailed search strategies for all databases are presented in Supplementary Table S1.

On 14 November 2021, we updated the search using the same search strategies.

### Inclusion Criteria

#### Types of Studies

We included randomized controlled trials (RCTs).

#### Types of Participants

We included participants of any age or gender with a primary clinical diagnosis of AIS.

#### Types of Interventions

The intervention group received XNJ combined with CTs, and the control group received the same CTs. Given the unclear overall scope of CTs, and in accordance with the AHA/ASA guidelines for early management of AIS (Powers et al., 2019) and the Chinese guidelines for diagnosis and treatments of AIS (Chinese Society of Neurology and Chinese Stroke Society, 2018), we decided to include trials using IVT, EVTs, antiplatelet treatment, statins, edaravone, or butylphthalide in consideration of the following factors:

1) In the Chinese guidelines, CTs are considered to also include anticoagulants, defibrases, neuroprotective agents, and other cerebral circulation improving drugs. In the AHA/ASA guidelines, however, class of recommendation (COR) Ⅰ only includes IVT, EVTs, and antiplatelet treatment. 2) For patients with AIS who qualify for statin treatment, in-hospital initiation or continuation of statin therapy is reasonable (COR Ⅱa); 3) Edaravone (Enomoto et al., 2019; Kobayashi et al., 2019) and dl-3-n-butylphthalide (NBP) (Wang et al., 2018; Xu et al., 2019) have extensive clinical foundation in China, and the latest established evidence indicated that they can not only improve the symptoms of ischemic stroke but also contribute to the long-term survival benefit.

#### Types of Outcomes

##### Efficacy Outcomes

The functional independence rate is used as the primary outcome. We defined functional independence as an mRS score of 0 to 2.

Secondary outcomes include neurologic deficit score (NDS), activities of daily living (ADL and Barthel Index), interleukin-6 (IL-6), tumor necrosis factor-alpha (TNF-α), high-sensitivity C-reactive protein (hs-CRP), and matrix metallopeptidase-9 (MMP-9). There were three evaluation criteria for NDS – the National Institutes of Health Stroke Scale (NIHSS), the Chinese Stroke Scale (CSS), and the European Stroke Scale (ESS).

##### Safety Outcomes

Safety outcomes include incidence of adverse reactions and adverse events.

### Exclusion Criteria

We excluded trials with the following features, in which: 1) other TCM treatments were applied in either the intervention or control group. 2) Outcomes were solely biochemical (for example, inflammatory markers) and not patient-centric (for example, the functional independence or NDS). 3) Outcomes were solely composite (for example, total effective rate) and not original (for example, NDS). 4) Outcomes were unclear (for example, NDS without definite evaluation criteria). 5) The course of treatment was less than 10 days or unclear. 6) The sample size was less than 60. 7) The language was not Chinese or English. 8) The full text was not available.

### Study Selection

Two reviewers (LDW and XMF) independently performed literature selection according to the predefined eligibility criteria. The records retrieved in all databases were imported into NoteExpress 3.2, and the duplicate records were deleted. The records were first screened based on the title and abstract, and in cases of uncertainty, the full texts were obtained. Any disagreement between the paired reviewers was resolved by discussing with a third reviewer (WS).

### Data Extraction

Two reviewers (LDW and YFC) independently extracted data from each trial using a predetermined data extraction form and then cross-checked data. Discrepancies were solved by discussion between the two reviewers or arbitrated by a third reviewer (WS).

We extracted the following data: 1) publication information (authors, country, and year of publication); 2) study designs (methods of randomization, allocation concealment, and blinding); 3) participant baseline characteristics and sample size; 4) details of intervention and control groups; and 5) outcomes (dichotomous data were number of events and total subjects per group; continuous data were mean, standard deviation, and total subjects per group). In case of missing data or unclear information, we contacted the original authors to clarify the information.

### Risk of Bias Assessment

Two reviewers (LDW and XMF) independently assessed the risk of bias of the included trials. We used the Cochrane Risk of Bias Tool 2.0 (Sterne et al., 2019) to evaluate the following five domains: randomization process, deviations from intended interventions, missing outcome data, outcome measurements, and selective reporting. Each domain was judged as either “low risk of bias”, “some concerns”, or “high risk of bias”. If disagreements on the judgment were identified, a third reviewer (WS) was consulted.

### Data Analysis

Review Manager (RevMan 5.4) software was utilized to perform data analyses. Risk ratio (*RR*) was used for dichotomous data, while weighted mean difference (*WMD*) or standardized mean difference (*SMD*) was used for continuous variables, and all of which were demonstrated with effect size and 95% confidence intervals (*CIs*). A fixed-effects model was selected when no significant heterogeneity was identified (*p* ≥ 0.10, or *I* ^2^ ≤ 50%). Otherwise, a random-effects model was applied. We performed subgroup analyses based on the course of treatment and the initiation time of XNJ. When the heterogeneity was substantial (*p* < 0.10, or *I* ^2^ > 50%), sources of heterogeneity would be fully explored given the data were accurate.

We further performed sensitivity analyses based on methodological quality and forest plots. After removing different trials in turn, we successively re-examined the meta-analysis results of the remaining trials to assess whether the statistical difference and heterogeneity change. If the findings changed evidently, the full texts of these trials would be checked, and we would interpret the results carefully.

To detect publication bias, we planned to generate funnel plots for meta-analyses including at least ten trials.

### Certainty Assessment

Two reviewers (LDW and YFC) independently assessed the certainty of the evidence using the Grading of Recommendations Assessment, Development and Evaluation (GRADE) approach (Balshem et al., 2011), and the evidence was classified as “high,” “moderate,” “low,” or “very low”. The certainty can be downgraded for five limitations (risk of bias, consistency of effect, imprecision, indirectness, and publication bias) and upgraded for three reasons (large magnitude of an effect, dose–response gradient, and effect of plausible residual confounding).

## Results

### Study Selection

**FIGURE 1:**
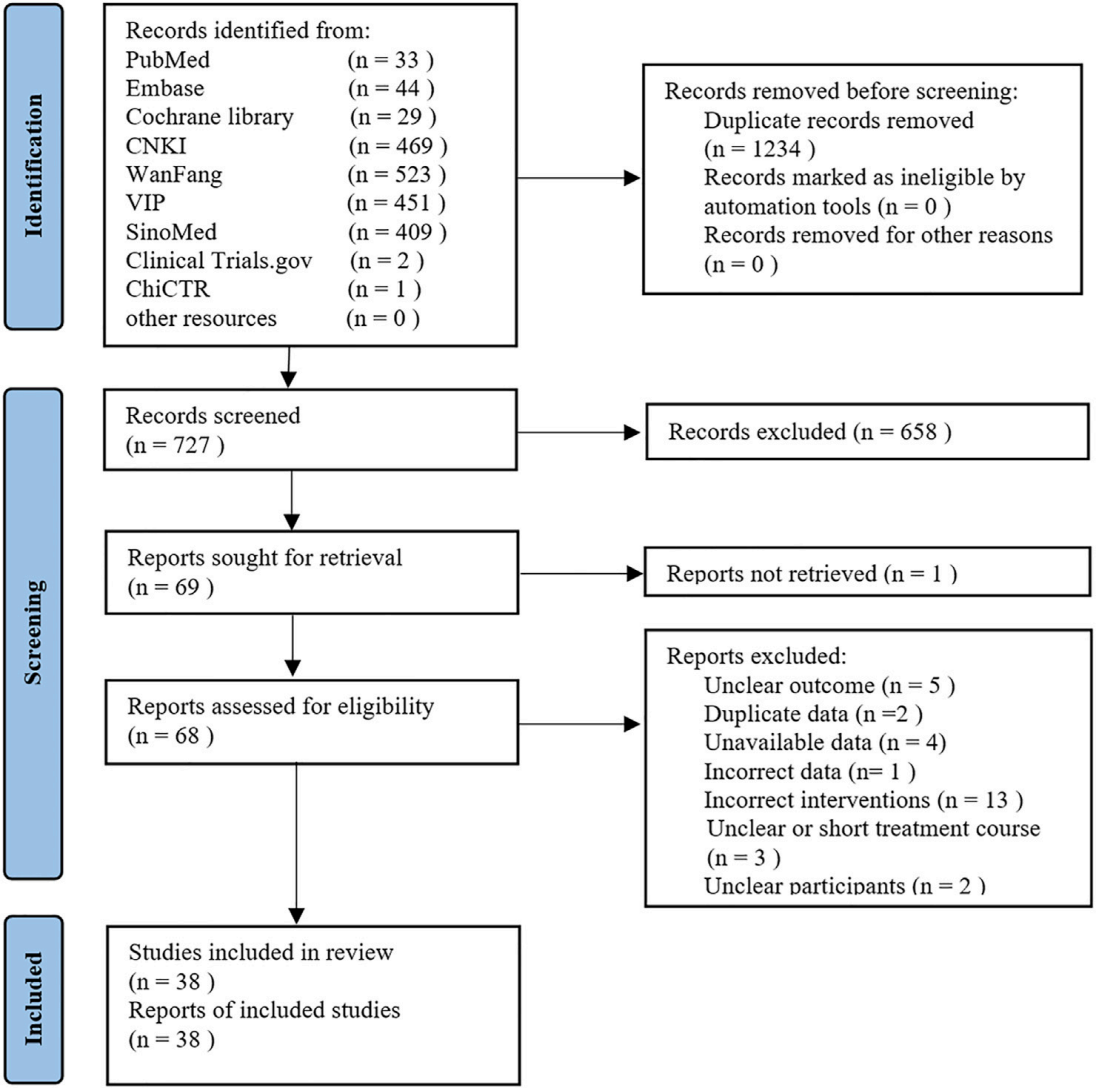
Flow diagram for identification of studies.

The search yielded 1961 records. There were 1,234 duplicates, leaving 727 to be screened by title and abstract, from which 69 eligible records were retained for full-text evaluation. After careful evaluation, 30 reports were excluded. Ultimately, 38 trials met our inclusion criteria (Wu, 2009; Guan, 2011; Chen et al., 2012; Li et al., 2012; Tong and Zhu, 2012; Zhang et al., 2012; Dong and Fu, 2013; Qian and Jia, 2013; Luo, 2014; Wei and Cheng, 2014; Chen and Wu, 2015; Yang and Li, 2015; Lu et al., 2016; Wei et al., 2016; Zhang and Ai, 2016; Feng et al., 2017; Ji et al., 2017; Shen et al., 2017; Wang et al., 2017; Wang and Lu, 2017; Wu et al., 2017; Zhao, 2017; Fu, 2018; Guo, 2018; Lin et al., 2018; Ma, 2018; Xiao et al., 2018; Yin and Liu, 2018; Liu C et al., 2019; Zheng et al., 2019; Liang et al., 2020; Liu, 2020; Wu and Xu, 2020; Zhang, 2020; Zhao, 2020; Chen, 2021; Dong et al., 2021; He, 2021). Figure 1 shows details of trial selection. A list of 30 trials that appeared to meet the inclusion criteria but excluded is reported in Supplementary Table S2 along with citation and reasons for exclusion.

### Study Characteristics

Thirty-eight RCTs involving 3,677 participants were included in this review, which included a total of 1841 patients in the intervention group and 1836 in the control group. Sample sizes of trials published from 2009 to 2021 ranged from 60 to 208. All trials were conducted in China and reported in Chinese. All interventions were XNJ in combination with CTs. Eight trials (Wei and Cheng, 2014; Zhao, 2017; Guo, 2018; Xiao et al., 2018; Zheng et al., 2019; Zhang, 2020; Chen, 2021; Dong et al., 2021) initiated XNJ treatment within 6 hours of stroke onset, and thirteen trials (Li et al., 2012; Zhang et al., 2012; Qian and Jia, 2013; Wei and Cheng, 2014; Lu et al., 2016; Zhang and Ai, 2016; Wang and Lu, 2017; Wu et al., 2017; Ma, 2018; Yin and Liu, 2018; Liang et al., 2020; Liu, 2020; He, 2021) initiated XNJ treatment within almost 1 day of stroke onset.

The proportion of functional independence at 14 days was reported by one trial (Ji et al., 2017). NDS was reported by all trials. Among them, 30 trials (Chen et al., 2012; Zhang et al., 2012; Qian and Jia, 2013; Wei and Cheng, 2014; Chen and Wu, 2015; Yang and Li, 2015; Lu et al., 2016; Zhang and Ai, 2016; Feng et al., 2017; Ji et al., 2017; Shen et al., 2017; Wang et al., 2017; Wang and Lu, 2017; Wu et al., 2017; Zhao, 2017; Guo, 2018; Lin et al., 2018; Ma, 2018; Xiao et al., 2018; Yin and Liu, 2018; Liu C et al., 2019; Zheng et al., 2019; Liang et al., 2020; Liu, 2020; Wu and Xu, 2020; Zhang, 2020; Zhao, 2020; Chen, 2021; Dong et al., 2021; He, 2021) adopted NIHSS, seven trials (Wu, 2009; Li et al., 2012; Tong and Zhu, 2012; Dong and Fu, 2013; Luo, 2014; Wei et al., 2016; Fu, 2018) adopted CSS, and four trials (Guan, 2011; Wei et al., 2016; Zheng et al., 2019; Liu, 2020) adopted ESS. The composition, source, and chemical characteristics of XNJ used in the included trials are presented in Supplementary Table S3. The other details are shown in Table 1.

**TABLE 1:** Basic characteristics of the included trials.

Study	Sample size		Male/female		Age/(year)		Course of disease/(day or hour)		Intervention group		Control group	Duration/(day)	Outcomes	
	T	C	T	C	T	C	T	C	XNJ	Combined with treatment			Efficacy	Safety
He, (2021)	47	47	24/23	25/22	58.24 ± 8.34	58.46 ± 6.79	(4–24) h	(4–25) h	Xingnaojing injection 10 ml/d	CTs	CTs	30	2) 8)	-
Dong et al. (2021)	62	62	32/30	33/29	58.59 ± 2.27	58.61 ± 2.31	(0–4.5) h		Xingnaojing injection 20 ml/d	CTs	CTs	14	2)	10) 11)
Chen, (2021)	45	45	20/25	22/23	60.4 ± 6.8	59.6 ± 6.5	(2.4 ± 0.9) h	(2.2 ± 0.8) h	Xingnaojing injection 20 ml/d	CTs	CTs	14	2)	-
Wu and Xu, (2020)	50	50	28/22	30/20	61.33 ± 9.81	60.86 ± 10.63	(1.36 ± 0.61) d	(1.45 ± 0.67) d	Xingnaojing injection 20 ml/d	CTs	CTs	14	2) 5) 6) 7) 8)	-
Zhao, (2020)	47	46	23/24	23/23	62.79 ± 9.34	62.39 ± 10.43	(0–14) d		Xingnaojing injection 20 ml/d	CTs	CTs	14	2) 6) 8)	10) 11)
Zhang, (2020)	30	30	18/12	17/13	64.81 ± 6.94	64.89 ± 6.97	(3.45 ± 0.86) h	(3.48 ± 0.85) h	Xingnaojing injection 20 ml/d	CTs	CTs	14	2)	10) 11)
Liu, (2020)	45	45	31/14	27/18	66.69 ± 6.98	67.42 ± 8.02	(0–24) h		Xingnaojing injection 20 ml/d	CTs	CTs	30	2) 4) 5)	10) 11)
Liang et al. (2020)	43	43	29/14	27/16	59.49 ± 4.92	59.37 ± 5.28	(13.92 ± 2.65) h	(13.85 ± 2.17) h	Xingnaojing injection 10–20 ml/d	CTs	CTs	14	2) 5) 6) 7) 8)	10) 11)
Liu H et al. (2019)	42	42	26/16	24/18	51.28 ± 6.14	51.69 ± 6.20	(0–72)		Xingnaojing injection 20 ml/d	CTs	CTs	14	2) 5)	-
Zheng et al. (2019)	43	43	26/17	27/16	59.74 ± 7.97	60.12 ± 8.23	(3–4) h		Xingnaojing injection 20 ml/d	CTs	CTs	14	2) 4) 5)	11)
Yin and Liu, (2018)	45	45	24/21	25/20	61.3 ± 12.70	60.8 ± 13.20	(23 ± 2.7) h	(24 ± 2.2) h	Xingnaojing injection 40 ml/d	CTs	CTs	14	2)	-
Ma, (2018)	43	43	19/24	21/22	57.76 ± 3.32	61.19 ± 3.71	(18.76 ± 3.32) h	(19.46 ± 4.79) h	Xingnaojing injection 20 ml/d	CTs	CTs	14	2)	10) 11)
Xiao et al. (2018)	70	70	48/22	47/23	-	-	(4.65 ± 0.19) h	(4.49 ± 0.31) h	Xingnaojing injection 10–20 ml/d	CTs	CTs	14	2) 6) 7) 8)	11)
Guo, (2018)	60	60	36/24	35/25	64.2 ± 12.63	63.3 ± 12.24	(4.4 ± 0.49) h	(4.3 ± 0.58) h	Xingnaojing injection 20 ml/d	CTs	CTs	10	2) 8) 9)	-
Fu, (2018)	37	37	25/12	23/14	65.36 ± 6.21	65.41 ± 6.17	-		Xingnaojing injection 20 ml/d	CTs	CTs	14	3)	10) 11)
Lin et al. (2018)	104	104	52/52	53/51	62.1 ± 5.3	61.3 ± 5.6	(0–72) h		Xingnaojing injection 20 ml/d	CTs	CTs	14	2)	10) 11)
Ji et al. (2017)	63	63	30/33	35/28	62.4 ± 4.9	61.6 ± 4.5	(24–120) h		Xingnaojing injection 20 ml/d	CTs	CTs	14	1) 2) 5)	-
Wang and Lu, (2017)	49	49	27/22	26/23	67.41 ± 6.25	67.93 ± 6.14	-		Xingnaojing injection 20 ml/d	CTs	CTs	14	2) 5) 6) 7) 8) 9)	-
Wu et al. (2017)	50	50	26/24	25/25	63.1 ± 3.4	64.2 ± 3.6	(0–24) h		Xingnaojing injection 20 ml/d	CTs	CTs	30	2) 5)	11)
Feng et al. (2017)	45	45	23/22	24/21	64.14 ± 7.24	64.72 ± 9.21	(0–72) h		Xingnaojing injection 20 ml/d	CTs	CTs	14	2) 5) 6) 7)	10) 11)
Wang et al. (2017)	35	35	18/17	19/16	60.2 ± 6.2	61.7 ± 5.3	(18.1 ± 6.2) h	(16.5 ± 5.9) h	Xingnaojing injection 30 ml/d	CTs	CTs	14	2) 5)	11)
Zhao, (2017)	31	29	21/10	19/10	71.02 ± 7.31	70.52 ± 7.41	(4.4 ± 0.49) h	(4.3 ± 0.58) h	Xingnaojing injection 20 ml/d	CTs	CTs	14	2) 5)	11)
Shen et al. (2017)	43	43	27/16	26/17	63.28 ± 6.48	62.54 ± 6.19	(1–3) d		Xingnaojing injection 20 ml/d	CTs	CTs	14	2) 5)	10) 11)
Lu et al. (2016)	35	35	18/17	19/16	60.1 ± 8.5	59.9 ± 8.7	(0–1) d		Xingnaojing injection 30 ml/d	CTs	CTs	14	2)	-
Zhang and Ai, (2016)	56	56	79/33		59 ± 9		(23 ± 3) h		Xingnaojing injection 30 ml/d	CTs	CTs	14	2)	-
Wei et al. (2016)	90	90	59/31	56/34	65 ± 5	64 ± 5	(2.2 ± 0.8) h	(2.4 ± 0.7) h	Xingnaojing injection 20 ml/d	CTs	CTs	14	3) 4)	-
Chen and Wu, (2015)	56	56	29/27	30/26	68.8 ± 7.3	68.3 ± 7.1	-		Xingnaojing injection 20 ml/d	CTs	CTs	21	2) 9)	-
Yang and Li, (2015)	58	58	33/25	37/21	63.7 ± 6.5	62.0 ± 6.1	(0–48) h		Xingnaojing injection 20 ml/d	CTs	CTs	14	2)	11)
Luo, (2014)	60	60	35/25	38/22	60.2 ± 10.2	61.7 ± 11.2	(38.12 ± 4.54) h	(41.58 ± 6.23) h	Xingnaojing injection 20 ml/d	CTs	CTs	14	3) 5)	11)
Wei and Cheng, (2014)	30	30	-	-	-	-	(6–24) h		Xingnaojing injection 20 ml/d	CTs	CTs	14	2)	11)
Qian and Jia, (2013)	40	40	19/21	16/24	61.2 ± 5.9	61.6 ± 6.3	(0–24) h		Xingnaojing injection 20 ml/d	CTs	CTs	14	2) 6)	-
Dong and Fu, (2013)	35	33	21/14	19/14	58.74 ± 7.63	59.2L ± 7.95	-		Xingnaojing injection 20 ml/d	CTs	CTs	14	3)	-
Li et al. (2012)	30	31	15/15	13/18	62	63	(0–24) h		Xingnaojing injection 20 ml/d	CTs	CTs	14	3)	11)
Zhang et al. (2012)	33	32	20/13	18/14	68.7 ± 10.5	69.2 ± 10.8	(0–24) h		Xingnaojing injection 20 ml/d	CTs	CTs	14	2)	-
Tong and Zhu, (2012)	64	66	38/26	40/26	61–85	62–84	(0–72) h		Xingnaojing injection 30 ml/d	CTs	CTs	14	3)	11)
Chen et al. (2012)	41	41	26/15	20/21	65.2 ± 12.6	63.5 ± 13.8	-		Xingnaojing injection 20 ml/d	CTs	CTs	14	2)	11)
Guan, (2011)	30	30	16/14	17/13	63.4 ± 10.7	61.4 ± 9.7	-		Xingnaojing injection 30 ml/d	CTs	CTs	14	4)	-
Wu, (2009)	54	52	30/24	30/22	63.45 ± 7.5	64.35 ± 7.36	(0–72) h		Xingnaojing injection 20 ml/d	CTs	CTs	14	3) 5) 6) 7)	-

XNJ, Xingnaojing injection; CTs, conventional treatments; C, control group; T, intervention group; 1), Functional independence rate; 2), NIHSS; 3), CSS; 4), ESS; 5), ADL; 6), IL-6; 7), TNF-α; 8), Hs-CRP; 9), MMP-9; 10), Incidence of adverse reactions; 11), Adverse events.

### Assessment of Risk of Bias

**FIGURE 2:**
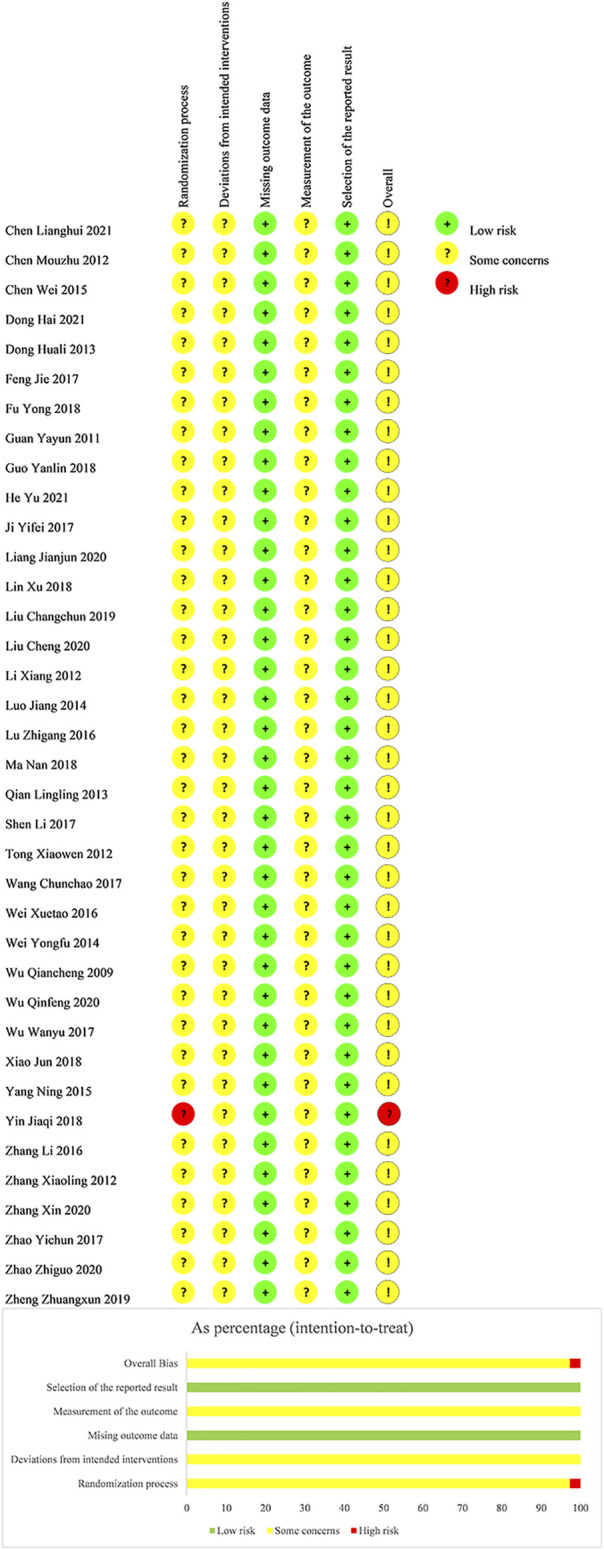
Risk of bias of included studies.

We summarized risk of bias of the included trials in Figure 2.

#### Domain 1: Risk of Bias Arising From the Randomization Process

For the generation of random sequence, 19 trials used a random number table (Zhang et al., 2012; Dong and Fu, 2013; Luo, 2014; Chen and Wu, 2015; Lu et al., 2016; Feng et al., 2017; Shen et al., 2017; Wang et al., 2017; Wang and Lu, 2017; Zhao, 2017; Fu, 2018; Guo, 2018; Lin et al., 2018; Liu H et al., 2019; Liang et al., 2020; Liu, 2020; Wu and Xu, 2020; Chen, 2021; He, 2021), one trial used a lottery (Dong et al., 2021), one trial used admission order (Yin and Liu, 2018), and the other seventeen trials lacked the adequate description of the randomization process (Wu, 2009; Guan, 2011; Chen et al., 2012; Li et al., 2012; Tong and Zhu, 2012; Qian and Jia, 2013; Wei and Cheng, 2014; Yang and Li, 2015; Wei et al., 2016; Zhang and Ai, 2016; Ji et al., 2017; Wu et al., 2017; Ma, 2018; Xiao et al., 2018; Zheng et al., 2019; Zhang, 2020; Zhao, 2020). No trial reported information regarding allocation concealment. Considering these, we judged 37 trials as “some concerns” and one trial (Yin and Liu, 2018) as “high risk of bias".

#### Domain 2: Risk of Bias due to Deviations From the Intended Interventions

As no information of blinding was present in any of the included trials, we judged all trials as “some concerns” with doubt of deviations from the intended interventions.

#### Domain 3: Risk of Bias due to Missing Outcome Data

As all outcome data were available, we judged all trials as “low risk of bias” in this domain.

#### Domain 4: Risk of Bias in Measurement of the Outcome

As no information of blinding assessors was present, we judged all trials as “some concerns".

#### Domain 5: Risk of Bias in Selection of the Reported Result

The planned outcome measurements and analyses in the *Method* section of published reports were fully reported without selection. Despite no available protocol, we judged all trials as “low risk of bias".

In view of the abovementioned evaluation, we judged the overall bias of one trial (Yin and Liu, 2018) as “high risk of bias” and other trials as “some concerns”.

### Efficacy Outcomes

#### Functional Independence Rate

**FIGURE 3:**
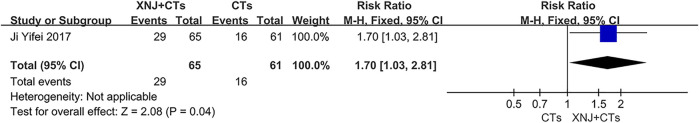
Forest plot of the functional independence rate.

One trial (Ji et al., 2017) determined the functional independence rate at 14 days after XNJ was initiated. The result demonstrated that the 14-day functional independence rate of XNJ plus CTs was higher than that of CTs alone (*RR* = 1.70, 95% *CI* = 1.03 to 2.81, *p* = 0.04; Figure 3).

#### NDS

Thirty-eight trials reported change of NDS at multiple time points including 14 days (Wu, 2009; Guan, 2011; Chen et al., 2012; Li et al., 2012; Tong and Zhu, 2012; Zhang et al., 2012; Dong and Fu, 2013; Qian and Jia, 2013; Luo, 2014; Wei and Cheng, 2014; Yang and Li, 2015; Lu et al., 2016; Wei et al., 2016; Zhang and Ai, 2016; Feng et al., 2017; Ji et al., 2017; Shen et al., 2017; Wang et al., 2017; Wang and Lu, 2017; Zhao, 2017; Fu, 2018; Guo, 2018; Lin et al., 2018; Ma, 2018; Xiao et al., 2018; Liu H et al., 2019; Zheng et al., 2019; Liang et al., 2020; Wu and Xu, 2020; Zhang, 2020; Zhao, 2020; Chen, 2021; Dong et al., 2021), 21 days (Chen and Wu, 2015), 28 days (Yin and Liu, 2018), and 30 days (Wu et al., 2017; Liu, 2020; He, 2021). According to the different time points, a subgroup analysis was conducted under the same evaluation criterion of NDS.

##### Scale 1: NIHSS

**FIGURE 4:**
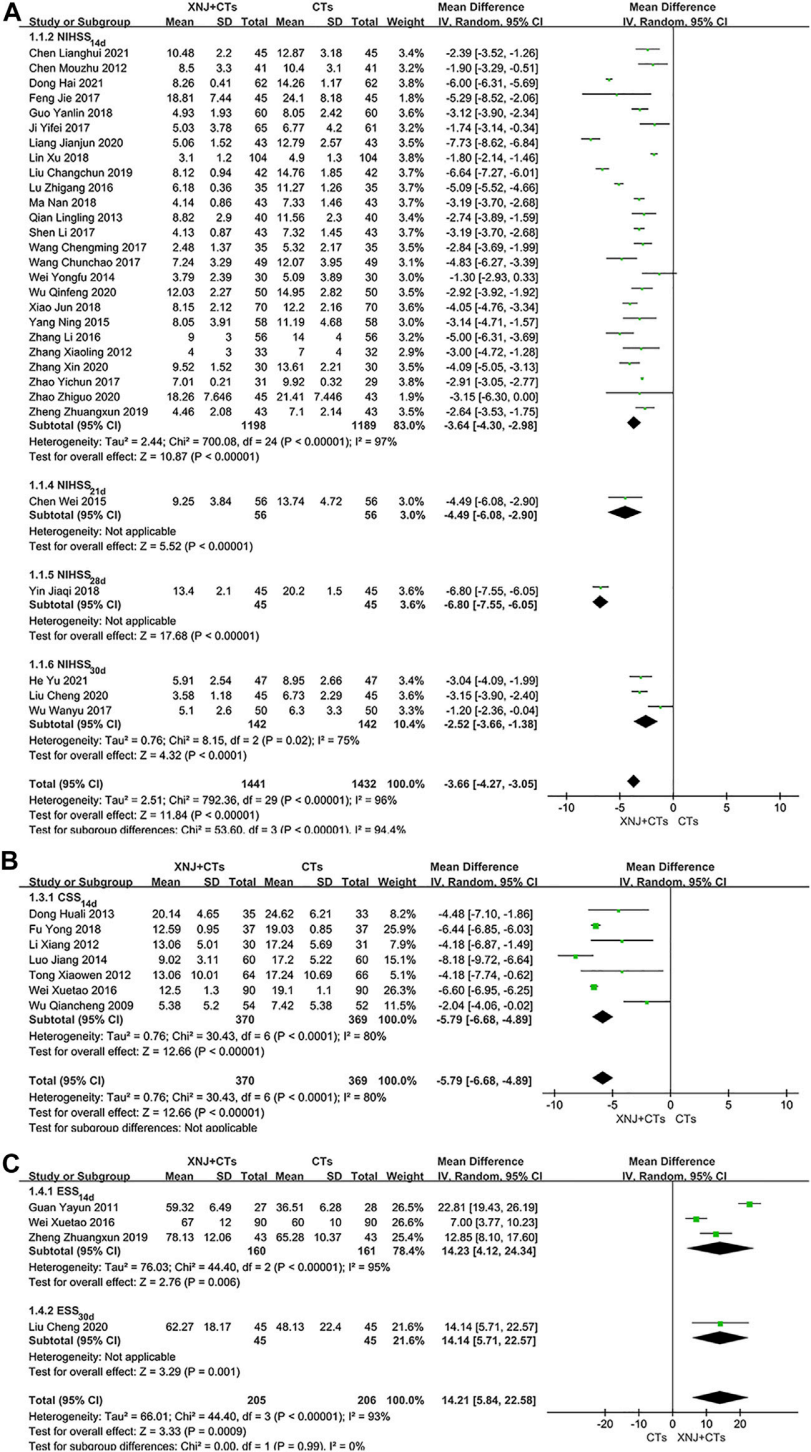
Forest plot of NIHSS **(A)**, CSS **(B),** and ESS **(C)**.

In total, thirty trials assessed NDS using NIHSS. The statistical heterogeneity among them was substantial (*I* ^2^_NIHSS-14d_ = 97%, *p* < 0.00001; *I* ^2^_NIHSS-30d_ = 75%, *p* = 0.02), so the random-effects model was used. As shown in the results, statistical difference was found between the two groups, which meant XNJ plus CTs was related to a more significant neurological recovery in NIHSS than CTs alone (*MD*_NIHSS-14d_ = −3.64, 95% *CI* = −4.30 to −2.98, *p* < 0.00001; *MD*_NIHSS-21d_ = −4.49, 95% *CI* = −6.08 to −2.90, *p* < 0.00001; *MD*_NIHSS-28d_ = −6.80, 95% *CI* = −7.55 to −6.05, *p* < 0.00001; *MD*_NIHSS-30d_ = −2.52, 95% *CI* = −3.66 to −1.38, *p* < 0.0001; Figure 4).

In the subgroup of the 14-day treatment course, we further performed a subgroup analysis according to the initiation time of XNJ. With the exception of two trials without specific course of disease (Chen et al., 2012; Wang et al., 2017), we divided 23 trials into four subgroups including within 6 hours (Zhao, 2017; Guo, 2018; Xiao et al., 2018; Zheng et al., 2019; Zhang, 2020; Dong et al., 2021) of stroke onset, 24 h (Zhang et al., 2012; Qian and Jia, 2013; Lu et al., 2016; Wei et al., 2016; Zhang and Ai, 2016; Wang and Lu, 2017; Ma, 2018; Liang et al., 2020; Chen, 2021), 72 h (Yang and Li, 2015; Feng et al., 2017; Shen et al., 2017; Lin et al., 2018; Liu H et al., 2019; Wu and Xu, 2020), and 14 days (Ji et al., 2017; Zhao, 2020). Despite the still significant heterogeneity (*I* ^2^_NIHSS < 6 h_ = 98%, *p* < 0.00001; *I* ^2^_NIHSS < 24h_ = 94%, *p* < 0.00001; *I* ^2^_NIHSS < 72h_ = 97%, *p* < 0.00001), the results demonstrated that the effect size was largest for the 6-h subgroup and smallest for the 14-day subgroup (*MD*_NIHSS < 6h_ = −3.81, 95% *CI* = −5.25 to −2.38, *p* < 0.00001; *MD*_NIHSS < 24h_= −3.75, 95% *CI* = −4.92 to −2.59, *p* < 0.00001; *MD*_NIHSS < 72 h_ = −3.74, 95% *CI* = −5.48 to −2.00, *p* < 0.0001; *MD*_NIHSS < 14 d_ = −1.97, 95% *CI* = −3.25 to −0.69, *p* = 0.003; Supplementary Figure S1). This suggested that the optimum initiation time of XNJ for AIS might be within 72 h, particularly the initial 6 hours. To reduce the heterogeneity, we conducted sensitivity analysis according to the distribution of trial confidence intervals on the forest plot. After removing trials whose confidence intervals hardly overlapped with those of the other trials, the heterogeneity was insignificant (*I* ^2^_NIHSS < 6 h_ = 45%, *p* = 0.16; *I* ^2^_NIHSS < 24 h_ = 14%, *p* = 0.32; *I* ^2^_NIHSS < 72 h_ = 0%, *p* = 0.89; Supplementary Figure S2). However, no obvious clinical heterogeneity was found in those removed trials.

##### Scale 2: CSS

A total of seven trials assessed NDS using CSS. The statistical heterogeneity was substantial (*I* ^2^_CSS-14d_ = 80%, *p* < 0.0001), so we used the random-effects model. The results showed that CSS in the XNJ group was lower than that in the control group with statistical significance (*MD*_CSS_ = −5.79, 95% *CI* = −6.68 to −4.89, *p* < 0.00001; Figure 4).

After a sensitivity analysis, we found that the exclusion of one trial (Wu, 2009) decreased heterogeneity by almost one-third (*I* ^2^_CSS-14d_ = 57%, *p* = 0.04). We re-visited the full text and concluded that the major source of heterogeneity was likely to be the difference in CSS before treatment. The improvement of XNJ on neurological function might be closely associated with the severity of the disease. The trial was removed, and the remaining six trials were pooled again (*MD*_CSS-14d_ = −6.44, 95% *CI* = −7.06 to −5.83, *p* < 0.00001; Supplementary Figure S3). We then performed a subgroup analysis based on the course of the disease. With the exception of two trials without a specific course of disease (Dong and Fu, 2013; Fu, 2018), we divided the remaining five trials into four subgroups including within 6 hours (Wei et al., 2016) of stroke onset, 24 h (Li et al., 2012), 48 h (Luo, 2014), and 72 h (Wu, 2009; Tong and Zhu, 2012). The results showed that the effect size of the 72-h subgroup was lower than those of the other subgroups with earlier XNJ initiated (*MD*_CSS < 6 h_ = −6.60, 95% *CI* = −6.95 to −6.25, *p* < 0.00001; *MD*_CSS < 24 h_ = −4.18, 95% *CI* = −6.87 to −1.49, *p* = 0.002; *MD*_CSS < 48 h_ = −8.18, 95% *CI* = −9.72 to −6.64, *p* < 0.00001; *MD*_CSS < 72 h_ = −2.59, 95% *CI* = −4.42 to −0.76, *p* = 0.006; Supplementary Figure S4). This correlation between the initiation time and efficacy is similar to that of NIHSS. However due to the small sample size, the optimal time of initiation requires further determination.

##### Scale 3: ESS

Four trials assessed NDS using ESS. Considering the substantial heterogeneity, we have applied the random-effects model for meta-analysis. The results showed that ESS in the XNJ group was higher than that of the control group with statistical significance (*MD*_ESS-14 d_ = 14.23, 95% *CI* = 4.12 to 24.34, *p* = 0.006; *MD*_ESS-30d_ = 14.14, 95% *CI* = 5.71 to 22.57, *p* = 0.001; Figure 4).

To explore the sources of statistical heterogeneity, we revisited the original literature and found that the initial ESS before treatment of three trials was inversely proportional to the increase of ESS. This clinical heterogeneity may result in the correspondingly large statistical heterogeneity and weaken the reliability of the results.

#### ADL–Barthel Score

**FIGURE 5:**
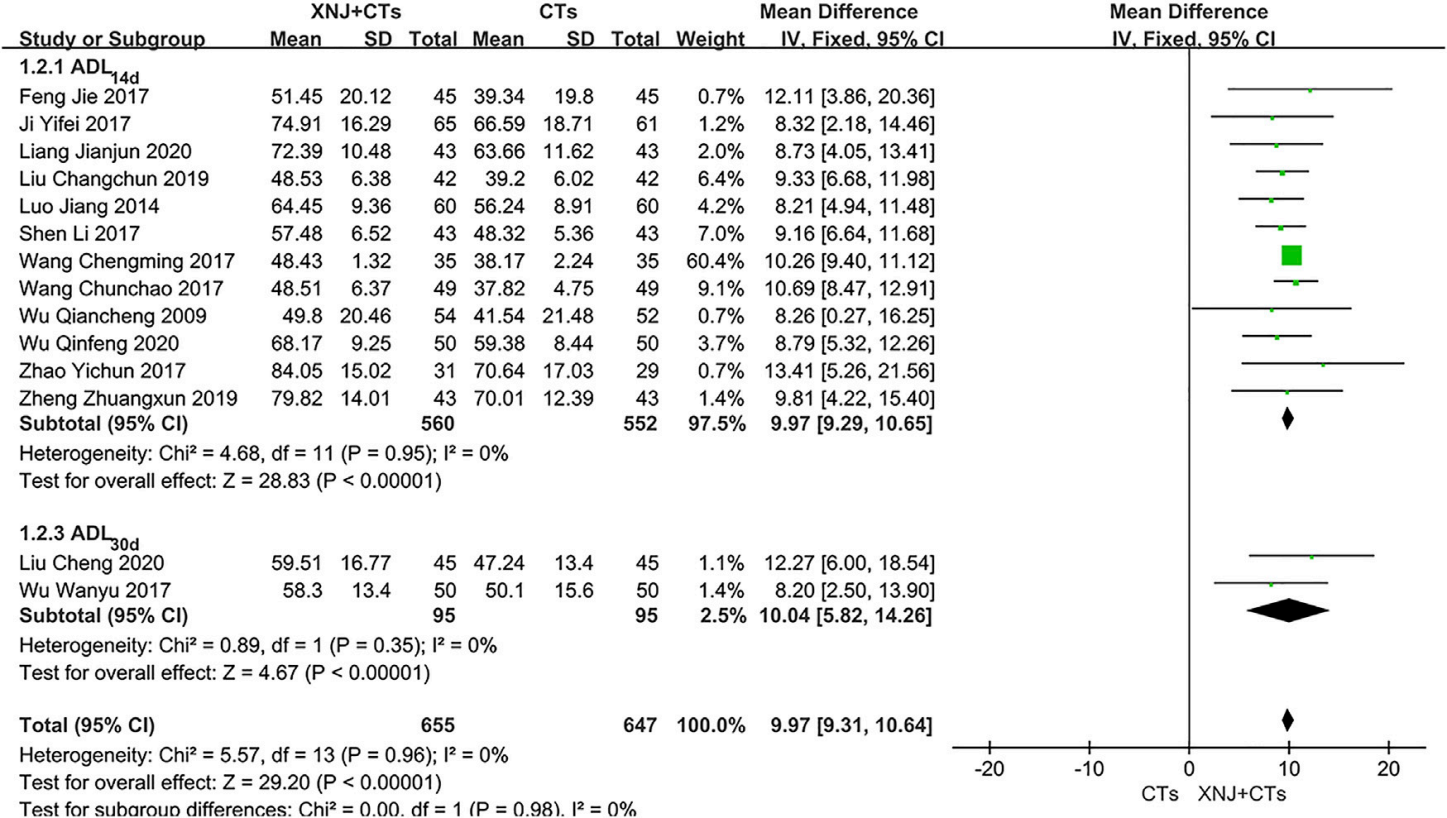
Forest plot of the ADL–Barthel score.

Fourteen trials observed the ADL–Barthel score. Subgroup analysis was carried out according to the different observation time points. There was no significant heterogeneity (*I* ^2^_BI-14d_ = 0%, *p* = 0.95; *I* ^2^_BI-30d_ = 0%, *p* = 0.35), so the fixed-effects model was used. The pooled results showed that XNJ plus CTs was superior to CTs alone in improving ADL with a statistical difference (*MD*_BI-14d_ = 9.97, 95% *CI* = 9.29 to 10.65, *p* < 0.00001; *MD*_BI-30d_ = 10.04, 95% *CI* = 5.82 to 14.26, *p* < 0.00001; Figure 5).

#### IL-6

**FIGURE 6:**
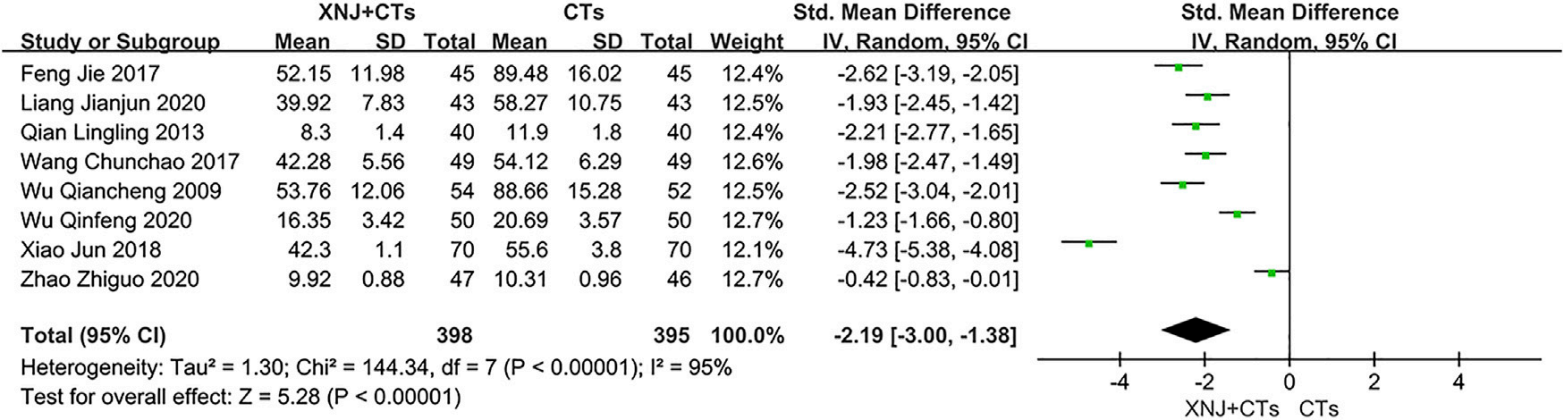
Forest plot of IL-6.

Eight trials reported IL-6. We adopted *SMD* because of the large difference in the mean among trials (Wen and Li, 2007). The statistical heterogeneity was significant (*I* ^2^ = 95%, *p* < 0.00001; Figure 6), and a random-effects model was used. Based on the use of *SMD* and large heterogeneity, we carried out descriptive analysis. All trials reported a statistical difference between the two groups, indicating that XNJ plus CTs is more effective than CTs alone in reducing IL-6.

Furthermore, a sensitivity analysis concluded that the differences in initial IL-6 may account for heterogeneity, and no other factors that could affect the heterogeneity were found. In the forest plot, three trials (Xiao et al., 2018; Wu and Xu, 2020; Zhao, 2020) had confidence intervals that hardly overlapped with those of the other five trials. We eliminated the three trials and pooled the remaining trials (*SMD*_IL-6_ = −2.23, 95% *CI* = −2.47 to −2.00, *p* < 0.00001; Supplementary Figure S5). Heterogeneity among the five trials was insignificant (*I* ^2^ = 25%, *p* = 0.26).

#### TNF-α

**FIGURE 7:**
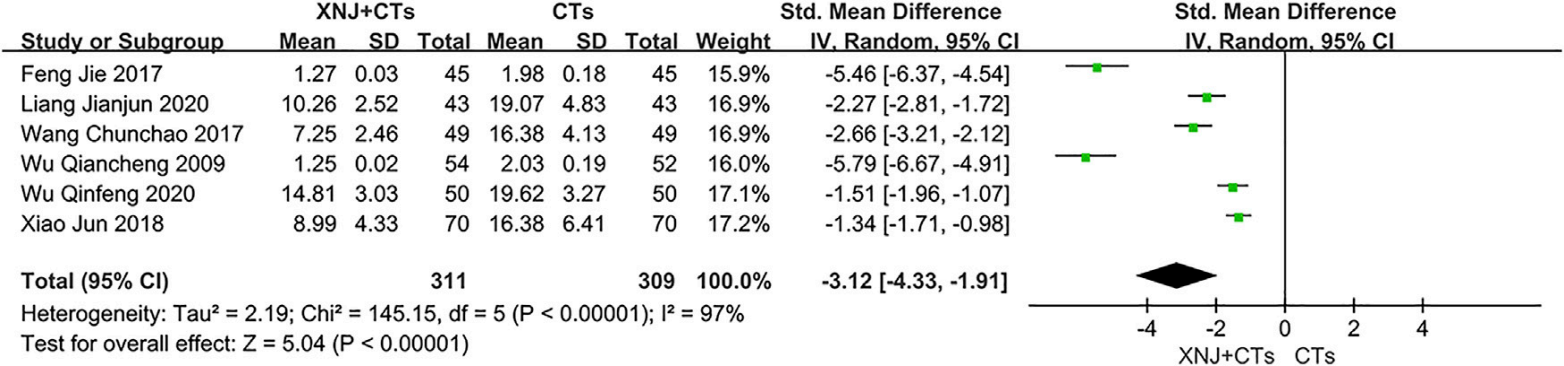
Forest plot of TNF-α.

Six trials reported TNF-α. We adopted *SMD* considering the large difference in the mean among trials. The statistical heterogeneity was substantial (*I* ^2^ = 97%, *p* < 0.00001; Figure 7), and therefore a random-effects model was used. We carried out descriptive analysis. All trials reported a statistically significant difference between the two groups, which supported the effectiveness of XNJ in reducing TNF-α.

#### Hs-CRP

**FIGURE 8:**
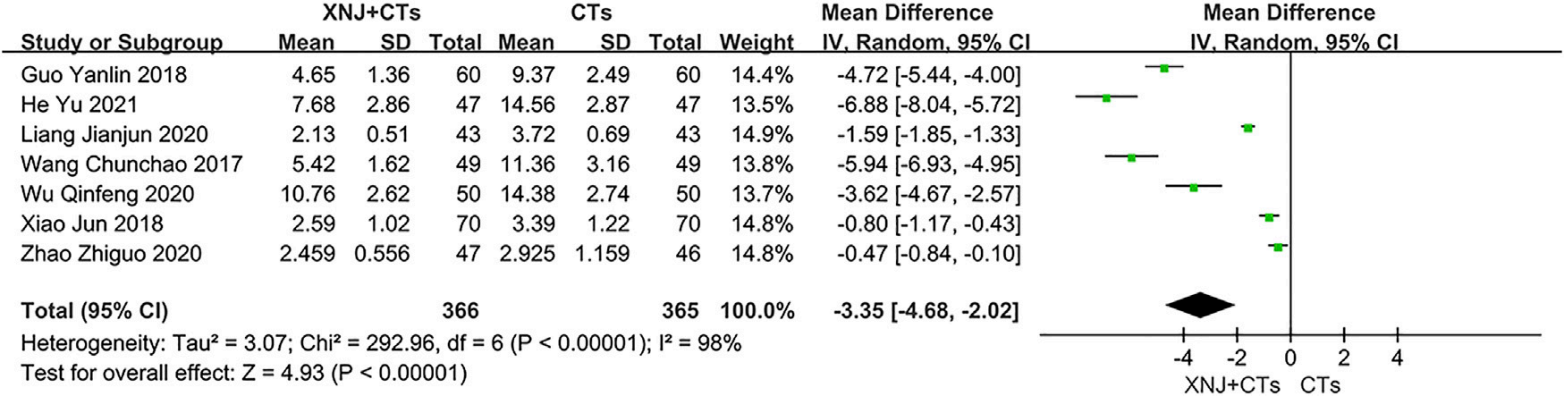
Forest plot of hs-CRP.

Seven trials reported hs-CRP. The heterogeneity among trials was substantial (*I* ^2^ = 98%, *p* < 0.00001; Figure 8), so a random-effects model was used. In addition to the baseline level of hs-CRP before treatment, no other factors that could cause such heterogeneity were found. We carried out descriptive analysis. All trials reported that XNJ plus CTs had a significant difference in reducing hs-CRP level compared with CTs alone.

#### MMP-9

**FIGURE 9:**
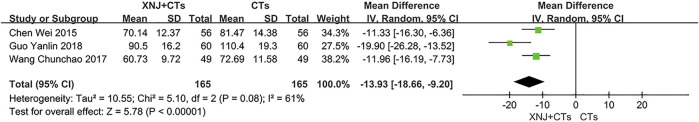
Forest plot of MMP-9.

Three trials reported MMP-9. The heterogeneity was moderate (*I* ^2^ = 61%, *p* = 0.08), and a random-effects model was used. There was a consequential difference. XNJ plus CTs, as compared with CTs alone, was associated with more significant decrease in MMP-9 (*MD*_MMP-9_ = −13.93, 95% *CI* = −18.66 to −9.20, *p* < 0.00001; Figure 9).

After a sensitivity analysis, we found that excluding one trial (Guo, 2018) can significantly reduce heterogeneity. Treatment course and baseline level of MMP-9 were likely the major sources of heterogeneity. The trial was then removed, and the other two trials were pooled again (*MD*_MMP-9_ = −11.69, 95% *CI* = −14.92 to −8.47, *p* < 0.00001; Supplementary Figure S6). Heterogeneity between the remaining two trials was insignificant (*I* ^2^ = 0%, *p* = 0.85).

### Safety Outcomes

#### Incidence of Adverse Reactions

**FIGURE 10:**
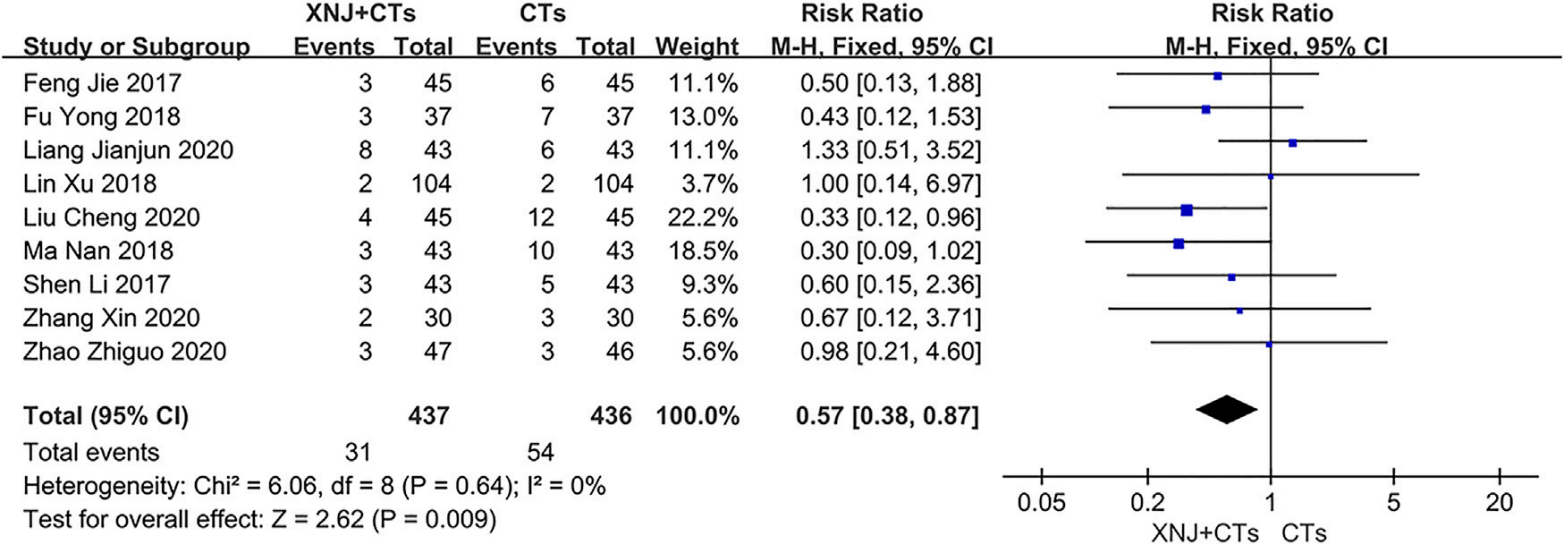
Forest plot of incidence of adverse reactions.

Nine trials reported the incidence of adverse reactions. Adverse reactions occurred in 31 out of 437 patients (7.1%) who received XNJ plus CTs and 54 out of 436 patients (12.4%) who received CTs alone. As the heterogeneity among the nine trials was insignificant (*I* ^2^ = 0%, *p* = 0.64), we used a fixed-effects model. The results showed that XNJ plus CTs incurred fewer incidences of adverse reactions than CTs alone with a statistical difference (*RR* = 0.57, 95% *CI* = 0.38 to 0.87, *p* = 0.009; Figure 10).

### Adverse Events

Twenty trials reported adverse events. Among them, nine studies (Li et al., 2012; Tong and Zhu, 2012; Luo, 2014; Wei and Cheng, 2014; Yang and Li, 2015; Wang and Lu, 2017; Wu et al., 2017; Zhao, 2017; Xiao et al., 2018) reported no serious adverse events in either group, and the other trials reported adverse events in both groups including gastrointestinal reactions, skin rashes, abnormal liver function, transient dizziness, slight headache, arrhythmia, dyspnea, gingival bleeding, and gastrointestinal bleeding. No participants discontinued the trial drug due to adverse events.

### Publication Bias

**FIGURE 11:**
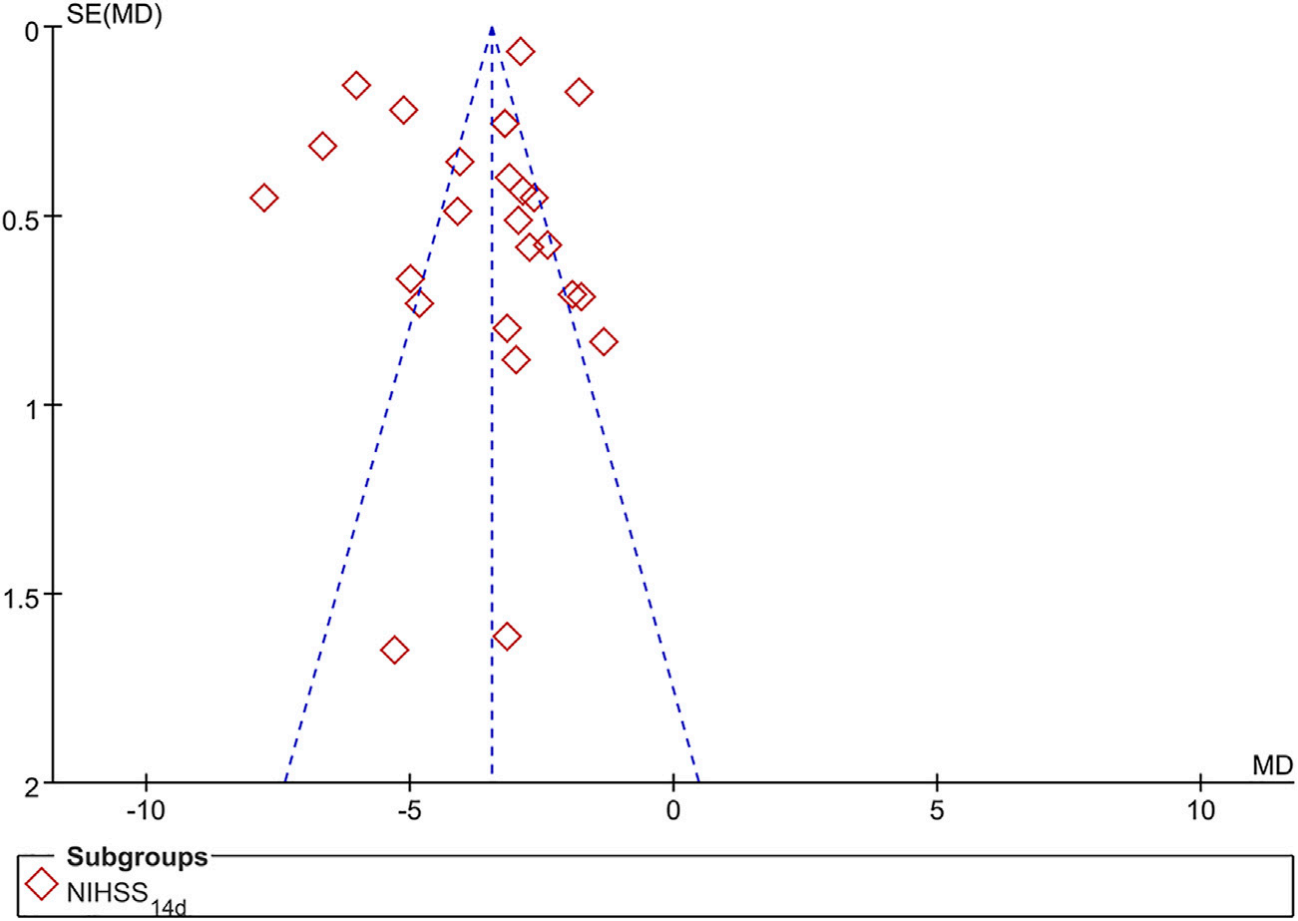
Funnel plot of NIHSS at 14 days.

The 14-day NIHSS of 24 trials was evaluated by the funnel plot, and the left–right asymmetry might be related to the Chinese publication of all included trials and unpublished negative results. As shown in Figure 11, the publication bias should be suspected to some extent.

### Sensitivity Analysis

Sensitivity analysis was conducted for all outcomes except for the functional independence rate. We omitted trials one-by-one in order to observe the meta-analysis result of the remaining trials. None of these exclusions altered the statistical significance of results, which indicated the robustness of our results. However, we found significant change in heterogeneity of CSS, IL-6, and MMP-9.

### GRADE Assessment

We used the GRADE approach to assess the quality of evidence for ten outcomes, and this ranged from “very low” to “low” with poor methodology, substantial heterogeneity, and publication bias. The certainty of evidence is summarized in Table 2.

**TABLE 2:** GRADE summary of outcomes for XNJ+CTs versus CTs for patients with AIS.

Outments	No. of participants (studies)	Anticipated absolute effects (95% *CI*)		Relative effect (95% *CI*)	Certainty of the evidence (GRADE)
		Risk with CTs	Risk difference with XNJ+CTs		
Functional independence rate (14d)	126 (1)	262 per 1,000	184 more per 1,000 (8 more to 475 more)	*RR* 1.70 (1.03–2.81)	⊕○○○ VERY LOW^a^ ^,^ ^b^ ^,^ ^c^
Incidence of adverse reactions	873 (9)	124 per 1,000	53 fewer per 1,000 (77 fewer to 16 more)	*RR* 0.57 (0.38–0.87)	⊕⊕○○ LOW^a^ ^,^ ^b^
NIHSS (14d)	2,387 (25)	The mean NIHSS (14d) ranged from 4.9 to 21.41	The mean NIHSS (14d) in the XNJI+CT group was 3.46 lower (3.56 lower to 3.36 lower)	-	⊕○○○ VERY LOW^a^ ^,^ ^b^ ^,^ ^d^
CSS (14d)	739 (7)	The mean CSS (14d) ranged from 7.42 to 24.62	The mean CSS (14d) in the XNJI+CT group was 5.79 lower (6.68 lower to 4.89 lower)	-	⊕○○○ VERY LOW^a^ ^,^ ^b^ ^,^ ^d^
ESS (14d)	321 (3)	The mean ESS (14d) ranged from 36.51 to 65.28	The mean ESS (14d) in the XNJI+CT group was 14.23 higher (4.12 higher to 24.34 higher)	-	⊕○○○ VERY LOW^a^ ^,^ ^b^ ^,^ ^d^ ^,^ ^e^
ADL–Barthel (14d)	1,112 (12)	The mean ADL–Barthel (14d) ranged from 37.82 to 70.64	The mean ADL–Barthel (14d) in the XNJI+CT group was 9.97 higher (9.29 higher to 10.65 higher)	-	⊕⊕○○ LOW^a^ ^,^ ^b^
IL-6	793 (8)	The mean IL-6 ranged from 10.31 to 89.48	The mean IL-6 in the XNJI+CT group was 2.19 lower (3.00 lower to 1.38 lower)	-	⊕○○○ VERY LOW^a^ ^,^ ^b^ ^,^ ^d^
TNF-α	620 (6)	The mean TNF-α ranged from 1.98 to 19.62	The mean TNF-α in the XNJI+CT group was 3.12 lower (4.33 lower to 1.91 lower)	-	⊕○○○ VERY LOW^a^ ^,^ ^b^ ^,^ ^d^
hs-CRP	731 (7)	The mean hs-CRP ranged from 2.93 to 14.56	The mean hs-CRP in the XNJI+CT group was 3.35 lower (4.68 lower to 2.02 lower)	-	⊕○○○ VERY LOW^a^ ^,^ ^b^ ^,^ ^d^
MMP-9	330 (3)	The mean MMP-9 ranged from 72.69 to 110.40	The mean MMP-9 in the XNJI+CT group was 13.93 lower (18.66 lower to 9.20 lower)	-	⊕○○○ VERY LOW^a^ ^,^ ^b^ ^,^ ^d^ ^,^ ^e^

XNJ, Xingnaojing injection; CTs, conventional treatments; CI, confidence interval; RR, relative risks.

a Poor methodology including method of randomization and blinding.

b Publication bias.

c Only one study provided data.

d *I* ^2^ ≥ 50% for heterogeneity.

e Small number of RCTs, with small sample sizes.

## Discussion

### Summary of Findings

In this review, we evaluated the efficacy and safety of XNJ as an emergency treatment for patients with AIS. We performed comprehensive literature search and identified 38 RCTs (3,677 participants). Compared with CTs alone, XNJ plus CTs was more effective for AIS in increasing the proportion of patients with independent function at 14 days (just one small sample trail), improving neurological function and restoring ability to perform daily activities.

Laboratory results showed positive effects of XNJ in improving IL-6, TNF-α, hs-CRP, and MMP-9. As a key cytokine in inflammatory response, IL-6 was found to be an independent risk predictor for AIS patients (Li et al., 2019), which may possibly be a new target in the prevention of short-term AIS death (Reiche et al., 2019). TNF-α, which plays an important role in the pathogenesis and the process of AIS, was also suggested to be a promising therapeutic target for the treatment of AIS (Wu et al., 2019). The anti-TNF therapy was found to be a feasible way to combat stroke disease *via* anti-inflammatory and metabolic mechanisms (Lin et al., 2021). Some studies (Huang et al., 2012; Vangilder et al., 2014) indicated that a high hs-CRP level was closely related to unfavorable long-term functional outcome and high rate of all-cause death 3 months after stroke. In addition, an elevated serum MMP-9 level in the acute phase of ischemic stroke was associated with increased risk of mortality and major disability, suggesting that serum MMP-9 could be an important prognostic factor for AIS (Zhong et al., 2017). Therefore, these outcomes are expected to be used as markers to predict the prognosis of AIS.

For safety evaluation, the results showed that the incidence of adverse reactions in the XNJ group was lower than that in the CT group. Nevertheless, it was worthy to note that both groups recorded slight gastrointestinal bleeding and arrhythmia. Although no difference in the incidence rate of these adverse reactions was observed between the two groups, it, nevertheless, suggested that close attention should be paid to the coagulation function and arrhythmia of patients after administering XNJ.

### Limitations of the Included Trials

The trials we included had certain limitations. First, all participants were from China only, and no data were available from other countries. Second, with regard to interventions, we were unable to objectively evaluate the efficacy of XNJ combined with or without the first-line emergency treatments as few trials described the situation of IVT and EVTs in patients. Third, with regard to outcomes, only one trial reported the proportion of functional independence, and the dependency was assessed only 2 weeks after the therapies were initiated. Thus, there was no sufficient evidence in improving the long-term function outcome. Finally, and most importantly, key methodological issues were present, including poor randomization procedures, unblinded design, and lack of necessary follow-up. None of the included trials reported allocation concealment or blinding, and bias in selection, performance, and detection reduced the reliability of the findings.

### Limitations of the Review

In conducting this review, we have come across certain limitations that might undermine the conclusiveness of the aforementioned findings. One of the major limitations was the substantial heterogeneity in NDS and biochemical outcomes, which affected the credibility of results. We speculated that the heterogeneity may have been resulted from the following factors:

1) Measurement of biochemical outcomes, with large fluctuation range, was easily influenced by different factors.
2) Clinical heterogeneity was present in the subtypes of ischemic stroke and the course of the disease.
3) Many trials failed to identify the syndrome of the subject, a concept of disease status in TCM.

When it comes to outcomes, most trials reported the total effective rate as a primary outcome, which we have chosen to exclude due to large uncertainty and non-standard evaluation of composite outcomes (Freemantle et al., 2003; Zhang Y Y et al., 2020). In spite of only one trial reporting the functional independence rate, we still used it as the primary outcome. The mRS has evolved as the primary outcome measure for acute stroke trials, and its application has demarcated effective and ineffective acute stroke therapies (Broderick et al., 2017; Powers, 2020). However, we excluded trials that used the entire ordinal distribution of the mRS as we contended that mRS measured by continuous statistical approach could affect the efficacy evaluation of overall functional independence due to a few severe cases. The incidences of death, hemorrhage, and other severe events should also be evaluated. In addition, the interventions included were different from the actual medical environment, which reduced the external validity.

### Implications for Future Research

As a consequence of the abovementioned problems, we put forward the following suggestions to future researchers.

1) For the exploration of heterogeneity in TCM trials, proper recording of the subjects’ syndrome (证型) will allow better explanation of heterogeneity. In the TCM theory, syndrome is an indispensable part of “syndrome differentiation and treatment (辨证论治)”.
2) For the selection of primary outcomes, researchers are encouraged to assess good functional outcome using 90-day mRS in order to more accurately and intuitively measure clinical benefit. As accepted by the U.S. Food and Drug Administration, the dichotomous approach in which mRS is divided into favorable and unfavorable outcomes should continue to be favored (Nunn et al., 2016). The length of follow-up should be at least 3 months (Lees et al., 2012). For phase II(b) trial, considering the time and resources required to measure mRS at 3 months and NIHSS within 1 week, a surrogate end point can also be used as a primary outcome endpoint (Chalos et al., 2020).
3) For the use of compound outcome the original NDS data should be provided when using the total effective rate to avoid exaggerating efficacy (Mccoy, 2018), and only used as a secondary outcome.
4) For the applicability of results, it would be important to also consider the real-world studies. Our team has been conducting real-world studies of XNJ for acute stroke, hoping to provide helpful empirical data for clinical decision-making.
5) Safety takes precedence over efficacy when evaluating XNJ combined with EVTs or IVT for AIS. A mixed-methods research (Tian, 2021) suggested that early use of XNJ within 6 hours of AIS onset was associated with greater functional improvement. However, the unclear timing of XNJ initiation is still a prominent problem largely due to medical insurance restrictions and concerns around possible conflict between XNJ and IVT. Fortunately, some randomized, parallel-group, double-blind, multicenter trials are ongoing, such as a trial of the Tiantan hospital to evaluate efficacy and safety of XNJ for AIS patients with endovascular thrombectomy and a trial of the Dongzhimen hospital to investigate whether XNJ can be initiated after reperfusion therapy or when AIS is not eligible for IVT (Lai et al., 2017).

Future research should be designed based on rigorous methodology, including allocation concealment, blinding, appropriate sample sizes, and longer follow-up assessment. Trial protocol should be registered on the website in advance, and the results should be reported according to the guidelines of SPIRIT-TCM Extension 2018 (Dai et al., 2019) and CONSORT-CHM Formulas 2017 (Cheng et al., 2017). We will be monitoring closely and updating this systematic review over time as high-quality evidence emerges.

## Conclusion

Initiating XNJ in the acute phase is effective in treating AIS. The optimum initiation time of XNJ for AIS might be the first 72 h after the onset of symptoms, in particular within the first 6 hours. However, due to insufficient evidence, it is inconclusive whether XNJ can be initiated immediately after the onset of AIS. However, due to the high risk of bias and substantial heterogeneity, the current evidence is not definitive. Given the low level of evidence, more rigorously designed and conducted RCTs, particularly those using the double-blind method, are warranted.

## Data Availability

The original contributions presented in the study are included in the article/Supplementary Material, further inquiries can be directed to the corresponding authors.

## References

[B1] Adogu P. Ubajaka C. F. Emelumadu O. F. Alutu C. (2015). Epidemiologic Transition of Diseases and Health-Related Events in Developing Countries: a Review. Am. J. Med. Med. Sci. 5 (4), 150–157. 10.5923/j.ajmms.20150504.02

[B2] Balshem H. Helfand M. Schünemann H. J. Oxman A. D. Kunz R. Brozek J. (2011). GRADE Guidelines: 3. Rating the Quality of Evidence. J. Clin. Epidemiol. 64 (4), 401–406. 10.1016/j.jclinepi.2010.07.015 21208779

[B3] Broderick J. P. Adeoye O. Elm J. (2017). Evolution of the Modified Rankin Scale and its Use in Future Stroke Trials. Stroke 48 (7), 2007–2012. 10.1161/STROKEAHA.117.017866 28626052 PMC5552200

[B4] Campbell B. C. V. Khatri P. (2020). Stroke. Lancet 396 (10244), 129–142. 10.1016/S0140-6736(20)31179-X 32653056

[B5] Chalos V. van der Ende N. A. M. Lingsma H. F. Mulder M. J. H. L. Venema E. Dijkland S. A. (2020). National Institutes of Health Stroke Scale: An Alternative Primary Outcome Measure for Trials of Acute Treatment for Ischemic Stroke. Stroke 51 (1), 282–290. 10.1161/STROKEAHA.119.026791 31795895 PMC6924951

[B6] Chen L. H. (2021). Effects of Xingnaojing Injection Combined with Intravenous Thrombolysis on Neurological Function, GFAP and MIF in Patients with Acute Ischemic Stroke. Chin. J. Integr. Med. Cardio/Cerebrovascuiar Dis. 19 (16), 2844–2847. CNKI:SUN:ZYYY.0.2021-16-033.

[B7] Chen M. Z. Chen Q. Y. Du F. Q. Xu S. Xiao Z. B. Gao B. R. (2012). Clinical Efficacy of Xingnaojing Injection Combined with Edaravone in the Treatment of Acute Ischemic Stroke. Seek Med. Ask Med. 10 (6), 496–497. CNKI:SUN:QYWA.0.2012-06-520.

[B8] Chen W. Wu X. B. (2015). Effect of Xingnaojing Injection on Serum Metalloproteinase-9, Nitric Oxide and Nitric Oxide Synthase in Patients with Acute Cerebral Infarction. Chin. J. Prim. Med. Pharm. 1 (20), 3061–3063. 10.3760/cma.j.issn.1008-6706.2015.20.007

[B9] Cheng C. W. Wu T. X. Shang H. C. Li Y. P. Altman D. G. Moher D. (2017). CONSORT Extension for Chinese Herbal Medicine Formulas 2017: Recommendations, Explanation, and Elaboration. Ann. Intern. Med. 167 (2), 112–121. 10.7326/M16-2977 28654980

[B10] China Food and Drug Administration (2003). National Drug Standards WS3-B-3353-98-2003. Beijing, China: China Food and Drug Administration.

[B11] Chinese Society of NeurologyChinese Stroke Society (2018). Chinese Guidelines for Diagnosis and Treatment of Acute Ischemic Stroke 2018. Chin. J. Neurol. 51 (9), 1. 10.3760/cma.j.issn.1006-7876.2018.09.004

[B12] Collaborators G. L. R. O. Feigin V. L. Nguyen G. Cercy K. Johnson C. O. Alam T. (2018). Global, Regional, and Country-specific Lifetime Risks of Stroke, 1990 and 2016. N. Engl. J. Me. 379 (25), 2429–2437. 10.1056/NEJMoa1804492 PMC6247346 30575491

[B13] Dai L. Cheng C. W. Tian R. Zhong L. L. Li Y. P. Lyu A. P. (2019). Standard Protocol Items for Clinical Trials with Traditional Chinese Medicine 2018: Recommendations, Explanation and Elaboration (SPIRIT-TCM Extension 2018). Chin. J. Integr. Med. 25 (1), 71–79. 10.1007/s11655-018-2999-x 30484022

[B14] Dong H. He Z. C. Liu L. (2021). To Explore the Clinical Effect of Xingnaojing Injection Combined with Alteplase Intravenous Thrombolysis in the Treatment of Acute Ischemic Stroke. J. Chengdu Med. Coll. 16 (05), 633–635.

[B15] Dong H. L. Fu J. L. (2013). Clinical Observation on Xingnaojing Injection in Treating Fever after Acute Cerebral Infarction. J. Emerg. Traditional Chin. Med. 22 (3), 469–470. 10.3969/j.issn.1004-745X.2013.03.069

[B16] Donkor E. S. (2018). Stroke in the 21st Century: A Snapshot of the Burden, Epidemiology, and Quality of Life. Stroke Res. Treat. 2018, 3238165. 10.1155/2018/3238165 30598741 PMC6288566

[B17] Enomoto M. Endo A. Yatsushige H. Fushimi K. Otomo Y. (2019). Clinical Effects of Early Edaravone Use in Acute Ischemic Stroke Patients Treated by Endovascular Reperfusion Therapy. Stroke 50 (3), 652–658. 10.1161/STROKEAHA.118.023815 30741623

[B18] Feigin V. L. Feigin V. L. Nguyen G. Cercy K. Johnson C. O. Alam T. (2018). Global, Regional, and Country-specific Lifetime Risks of Stroke, 1990 and 2016. N. Engl. J. Med. 379 (25), 2429–2437. 10.1056/NEJMoa1804492 30575491 PMC6247346

[B19] Feng J. Che F. Y. Zhang M. (2017). Clinical Observation of Xingnaojing Injection in the Treatment of Acute Cerebral Infarction. China Pharm. 28 (32), 4548–4551. 10.6039/j.issn.1001-0408.2017.32.23

[B20] Freemantle N. Calvert M. Wood J. Eastaugh J. Griffin C. (2003). Composite Outcomes in Randomized Trials: Greater Precision but with Greater Uncertainty? Jama 289 (19), 2554–2559. 10.1001/jama.289.19.2554 12759327

[B21] Fu C. Zhang X. Lu Y. Wang F. Xu Z. Liu S. (2020). Geniposide Inhibits NLRP3 Inflammasome Activation via Autophagy in BV-2 Microglial Cells Exposed to Oxygen-Glucose Deprivation/reoxygenation. Int. Immunopharmacol. 84, 106547. 10.1016/j.intimp.2020.106547 32361652

[B22] Fu Y. (2018). Clinical Effect of Xingnaojing Injection Combined with Alteplase in the Treatment of Acute Ischemic Stroke. J. Med. Forum 39 (1), 142–143. CNKI:SUN:HYYX.0.2018-01-056.

[B23] Guan Y. Y. (2011). Effect of Xingnaojing on Severe Cerebral Infarction Patients with Coma. Nei Mongol J. Traditional Chin. Med. 30 (8), 13–14. 10.3969/j.issn.1006-0979.2011.08.015

[B24] Guo Y. L. (2018). Clinical Study of Xingnaojing Injection Combined with Urokinase in the Treatment of Acute Cerebral Infarction. Clin. J. Chin. Med. 10 (13), 16–18. 10.3969/j.issn.1674-7860.2018.13.007

[B25] Hao Z. L. Liu M. Li W. Tan Y. Zhang Y. H. Wu Li. E. (2011). Basic Characteristics and Functional Outcomes of 3123 Consecutive Patients in Chengdu Stroke Registry. Chin. J. Neurol. 44 (12), 826–831. CNKI:SUN:ZHSJ.0.2011-12-012.

[B26] Hasnain M. G. Attia J. R. Akter S. Rahman T. Hall A. Hubbard I. J. (2020). Effectiveness of Interventions to Improve Rates of Intravenous Thrombolysis Using Behaviour Change Wheel Functions: a Systematic Review and Meta-Analysis. Implement. Sci. 15 (1), 98. 10.1186/s13012-020-01054-3 33148294 PMC7641813

[B27] He Y. (2021). Effects of Xingnaojing Injection Combined with Edaravone on Serum Lipoprotein Associated Phospholipase A2 and Erythropoietin in Patients with Acute Ischemic Stroke. China's Naturopathy. 29 (09), 88–91. 10.19621/j.cnki.11-3555/r.2021.0933

[B28] Huang Y. Jing J. Zhao X. Q. Wang C. X. Wang Y. L. Liu G. F. (2012). High-Sensitivity C-Reactive Protein Is a Strong Risk Factor for Death after Acute Ischemic Stroke Among Chinese. CNS Neurosci. Ther. 18 (3), 261–266. 10.1111/j.1755-5949.2012.00296.x 22449109 PMC6493374

[B29] Ji Y. F. Long J. F. Zhou H. Y. Ren H. Zhang Y. Du X. (2017). Clinical Effect of Xingnaojing Injection on Acute Cerebral Infarction. Guide China Med. 15 (13), 1–3. 10.15912/j.cnki.gocm.2017.13.001

[B30] Kobayashi S. Fukuma S. Ikenoue T. Fukuhara S. Kobayashi S. (2019). Effect of Edaravone on Neurological Symptoms in Real-World Patients with Acute Ischemic Stroke. Stroke 50 (7), 1805–1811. 10.1161/STROKEAHA.118.024351 31164072

[B31] Lai X. Cao K. Kong L. Liu Q. Gao Y. (2017). Xingnaojing for Moderate-To-Severe Acute Ischemic Stroke (XMAS): Study Protocol for a Randomized Controlled Trial. Trials 18 (1), 479. 10.1186/s13063-017-2222-y 29037226 PMC5644245

[B32] Lee D. Kim Y. S. Song J. Kim H. (2019). Neuroprotective Effects of Musk of Muskrat on Transient Focal Cerebral Ischemia in Rats. Evid. Based Complement. Alternat Med. 2019, 9817949–9817956. 10.1155/2019/9817949 31341507 PMC6614976

[B33] Lees K. R. Bath P. M. Schellinger P. D. Kerr D. M. Fulton R. Hacke W. (2012). Contemporary Outcome Measures in Acute Stroke Research: Choice of Primary Outcome Measure. Stroke 43 (4), 1163–1170. 10.1161/STROKEAHA.111.641423 22426313

[B34] Li X. Lin S. Chen X. Huang W. Li Q. Zhang H. (2019). The Prognostic Value of Serum Cytokines in Patients with Acute Ischemic Stroke. Aging Dis. 10 (3), 544–556. 10.14336/AD.2018.0820 31164999 PMC6538221

[B35] Li X. Li Y. H. Fang Y. Fang J. Li X. H. Huang R. (2012). Effect of Xingnaojing Injection on Acute Massive Cerebral Infarction. Chin. J. Pract. Nervous Dis. 15 (22), 76–78. 10.3969/j.issn.1673-5110.2012.22.044

[B36] Liang J. J. Tang R. Li H. Liang Y. Xu F. (2020). Clinical Study of Xingnaojing Injection Combined with Alteplase in the Treatment of Acute Ischemic Stroke. China Med. Herald 17 (2), 148–151. CNKI:SUN:YYCY.0.2020-02-037.

[B37] Lin S. Y. Wang Y. Y. Chang C. Y. Wu C. C. Chen W. Y. Liao S. L. (2021). TNF-α Receptor Inhibitor Alleviates Metabolic and Inflammatory Changes in a Rat Model of Ischemic Stroke. Antioxidants (Basel) 10 (6), 851. 10.3390/antiox10060851 34073455 PMC8228519

[B38] Lin X. Zhou J. H. Li S. H. Luo C. B. Li G. N. (2018). Effect of Xingnaojing Combined with Butylphthalide on Patients with Acute Cerebral Infarction and its Influence on Neurological Function. Anti-Infection Pharm. 15 (3), 392–395. 10.13493/j.issn.1672-7878.2018.03-007

[B39] Liu C. C. Xu T. Mo W. Y. Hao L. M. (2019). Effects of Xingnaojing Combined with Edaravone on Serum Oxidative Stress Indexes, Neurological Function and Hemodynamics in Patients with Massive Cerebral Infarction. Prev. Treat. Cardio-Cerebral-Vascular Dis. 19 (5), 458–460. 10.3969/j.issn.1009-816x.2019.05.024

[B40] Liu C. (2020). Effect of Xingnaojing Injection Combined with Alteplase Intravenous Thrombolysis on Neurological Function in Patients with Acute Cerebral Infarction. J. Clin. Res. 37 (1), 42–44. 10.3969/j.issn.1671-7171.2020.01.014

[B41] Liu F. Ying Y. Zhao R. Li J. Li P. Li Z. (2010). Establishment of the Guidelines for Assigned Xingnaojing Injection of Medical Insurance in Beijing. China Pharm. 1 (28), 2686–2688.

[B42] Liu H. Yan Y. Pang P. Mao J. Hu X. Li D. (2019). Angong Niuhuang Pill as Adjuvant Therapy for Treating Acute Cerebral Infarction and Intracerebral Hemorrhage: A Meta-Analysis of Randomized Controlled Trials. J. Ethnopharmacol. 237, 307–313. 10.1016/j.jep.2019.03.043 30910581

[B43] Liu Z. Ran Y. Huang S. Wen S. Zhang W. Liu X. (2017). Curcumin Protects against Ischemic Stroke by Titrating Microglia/Macrophage Polarization. Front. Aging Neurosci. 9, 233. 10.3389/fnagi.2017.00233 28785217 PMC5519528

[B44] Lu Z. G. Liu Y. Fang J. H. (2016). Effect of Xingnaojing Injection on Neurological Deficit in Patients with Acute Cerebral Infarction and its Mechanism. Chin. J. Integrated Traditional West. Med. Intensive Crit. Care 23 (4), 352–355. 10.3969/j.issn.1008-9691.2016.04.004

[B45] Luo J. (2014). Clinical Observation of Xingnaojing Injection Combined with Western Medicine in the Treatment of Acute Cerebral Infarction. J. Traditional Chin. Med. Univ. Hunan 1 (5), 52–54. 10.3969/j.issn.1674-070X.2014.05.015.052.03

[B46] Ma N. (2018). Clinical Effect of Xingnaojing Injection Combined with Alteplase Intravenous Thrombolysis in the Treatment of Patients with Acute Ischemic Stroke. Chin. J. Med. Device 31 (22), 133–134. 10.3969/j.issn.1002-2376.2018.22.098

[B47] Ma R. Ma X. Wen J. Wang J. Xie Q. Chen N. (2018). Preclinical Evidence and Mechanism of Xingnaojing Injection for Cerebral Ischemia: A Systematic Review and Meta-Analysis of Animal Studies. Evid. Based Complement. Alternat Med. 2018, 9624175. 10.1155/2018/9624175 30581490 PMC6276459

[B48] Ma X. Yang Y. X. Chen N. Xie Q. Wang T. He X. (2017). Meta-Analysis for Clinical Evaluation of Xingnaojing Injection for the Treatment of Cerebral Infarction. Front. Pharmacol. 8, 485. 10.3389/fphar.2017.00485 28912713 PMC5583602

[B49] Mccoy C. E. (2018). Understanding the Use of Composite Endpoints in Clinical Trials. West. J. Emerg. Med. 19 (4), 631–634. 10.5811/westjem.2018.4.38383 30013696 PMC6040910

[B50] Nunn A. Bath P. M. Gray L. J. (2016). Analysis of the Modified Rankin Scale in Randomised Controlled Trials of Acute Ischaemic Stroke: A Systematic Review. Stroke Res. Treat. 2016, 9482876–9482877. 10.1155/2016/9482876 27088036 PMC4818820

[B51] Page M. J. Mckenzie J. E. Bossuyt P. M. Boutron I. Hoffmann T. C. Mulrow C. D. (2021). The PRISMA 2020 Statement: An Updated Guideline for Reporting Systematic Reviews. J. Clin. Epidemiol. 134, 178–189. 10.1016/j.jclinepi.2021.03.001 33789819

[B52] Peng W. Yang J. Wang Y. Wang W. Xu J. Wang L. (2014). Systematic Review and Meta-Analysis of Randomized Controlled Trials of Xingnaojing Treatment for Stroke. Evid. Based Complement. Alternat Med. 2014, 210851–210859. 10.1155/2014/210851 24707306 PMC3953647

[B53] Pharmacopoeia Committee of the Ministry of Public Health of the People’s Republic of China (1998). Drug Standard of Ministry of Public Health of the People’s Republic of China - Chinese Patent Drugs. Beijing, China: Pharmacopoeia Committee of the Ministry of Public Health of the People’s Republic of China, 1.

[B54] Powers W. J. (2020). Acute Ischemic Stroke. N. Engl. J. Med. 383 (3), 252–260. 10.1056/NEJMcp1917030 32668115

[B55] Powers W. J. Rabinstein A. A. Ackerson T. Adeoye O. M. Bambakidis N. C. Becker K. (2019). Guidelines for the Early Management of Patients with Acute Ischemic Stroke: 2019 Update to the 2018 Guidelines for the Early Management of Acute Ischemic Stroke: A Guideline for Healthcare Professionals from the American Heart Association/American Stroke Association. Stroke 50 (12), e344. 10.1161/STR.0000000000000211 31662037

[B56] Qian L. L. Jia K. (2013). Effect of Xingnaojing Injection on Brain protection and Levels of Interleukin-6 and Interleukin-8 in Patients with Acute Cerebral Infarction. Chin. Traditional Patent Med. 35 (8), 1633–1636. 10.3969/j.issn.1001-1528.2013.08.010

[B57] Qu X. Y. Zhang Y. M. Tao L. N. Gao H. Zhai J. H. Sun J. M. (2019). XingNaoJing Injections Protect against Cerebral Ischemia/reperfusion Injury and Alleviate Blood-Brain Barrier Disruption in Rats, through an Underlying Mechanism of NLRP3 Inflammasomes Suppression. Chin. J. Nat. Med. 17 (7), 498–505. 10.1016/S1875-5364(19)30071-8 31514981

[B58] Reiche E. M. V. Gelinksi J. R. Alfieri D. F. Flauzino T. Lehmann M. F. de Araújo M. C. M. (2019). Immune-inflammatory, Oxidative Stress and Biochemical Biomarkers Predict Short-Term Acute Ischemic Stroke Death. Metab. Brain Dis. 34 (3), 789–804. 10.1007/s11011-019-00403-6 30875023

[B59] Sharobeam A. Jones B. Walton-Sonda D. Lueck C. J. (2021). Factors Delaying Intravenous Thrombolytic Therapy in Acute Ischaemic Stroke: a Systematic Review of the Literature. J. Neurol. 268 (8), 2723–2734. 10.1007/s00415-020-09803-6 32206899

[B60] Shen L. Lou Q. L. Ma H. F. (2017). Effect of Xingnaojing Injection Combined with Alteplase in the Treatment of Acute Ischemic Stroke and its Influence on Cognitive Function. New J. Tradit. Chin. Med. 49 (5), 14–16. 10.13457/j.cnki.jncm.2017.05.005

[B61] Sterne J. A. C. Savović J. Page M. J. Elbers R. G. Blencowe N. S. Boutron I. (2019). RoB 2: a Revised Tool for Assessing Risk of Bias in Randomised Trials. BMJ 366, l4898. 10.1136/bmj.l4898 31462531

[B62] Tao Y. R. Liu T. T. Sun F. L. Ai H. X. Guo D. Y. Wang W. (2018). Preclinical Research Progress of Enlarging Thrombolysis Time Window of Ischemic Stroke with Tissue Type Plasminogen Activator Treatment. Chin. J. Comp. Med. 28 (8), 118–123. 10.3969/j.issn.1671-7856.2018.08.021

[B63] Tian Z. Y. Feng L. D. Xie Y. Xu D. H. Zhang C. Y. Kong L. B. (2021). Chinese Herbal Medicine Xingnaojing Injection for Acute Ischemic Stroke: An Overview of Systematic Reviews and Meta-Analyses. Front. Pharmacol. 12, 659408. 10.3389/fphar.2021.659408 34084137 PMC8167030

[B64] Tian Z. Y. (2021). Evidence Based Evaluation of Early Intervention of Xingnaojing in Acute Stroke and a Mixed Method Study on the Effect on Patients' Prognosis. Beijing: Beijing University of Chinese Medicine.

[B65] Tong X. W. Zhu J. (2012). Effect of Xingnaojing Injection on Acute Cerebral Infarction in the Elderly. Mod. J. Integrated Traditional Chin. West. Med. 21 (4), 387–388. 10.3969/j.issn.1008-8849.2012.04.022

[B66] Vangilder R. L. Davidov D. M. Stinehart K. R. Huber J. D. Turner R. C. Wilson K. S. (2014). C-reactive Protein and Long-Term Ischemic Stroke Prognosis. J. Clin. Neurosci. 21 (4), 547–553. 10.1016/j.jocn.2013.06.015 24211144 PMC4394376

[B67] Wang C. C. Miao H. Sang H. C. Li G. L. (2017). Effect of Xingnaojing Injection on Serum Cytokines and Neurological Function in Patients with Acute Cerebral Infarction. Chin. J. Integr. Med. Cardio/Cerebrovascular Dis. 15 (14), 1772–1775. 10.3969/j.issn.1672-1349.2017.14.030

[B68] Wang C. M. Lu Z. G. (2017). Effect of Xingnaojing Injection on Blood Stasis Syndrome and Hemorheology in Elderly Patients with Acute Cerebral Infarction. Chin. J. Gerontol. 37 (12), 2910–2912. 10.3969/j.issn.1005-9202.2017.12.021

[B69] Wang S. Ma F. Huang L. Zhang Y. Peng Y. Xing C. (2018). Dl-3-n-Butylphthalide (NBP): a Promising Therapeutic Agent for Ischemic Stroke. CNS Neurol. Disord. Drug Targets 17 (5), 338–347. 10.2174/1871527317666180612125843 29895257

[B70] Wang Z. Li J. Wang C. Yao X. Zhao X. Wang Y. (2013). Gender Differences in 1-year Clinical Characteristics and Outcomes after Stroke: Results from the China National Stroke Registry. PLoS One 8 (2), e56459. 10.1371/journal.pone.0056459 23418571 PMC3572058

[B71] Wei X. T. Liu H. H. Ma R. J. (2016). Effects of Xingnaojing Combined with Alteplase on Neurological Function and Related Factors in the Treatment of Acute Ischemic Stroke. Hebei Med. J. 38 (14), 2155–2157. 10.3969/j.issn.1002-7386.2016.14.021

[B72] Wei Y. F. Cheng Y. H. (2014). Effect of Xingnaojing Combined with Butylphthalide on Neurological Function and Clinical Efficacy in the Treatment of Acute Cerebral Infarction. J. Med. Theor. Pract. 27 (4), 462–463. 10.19381/j.issn.1001-7585.2014.04.021

[B73] Wen J. Li Y. P. (2007). The Selection of a Summary Statistic for Use in Meta-Analysis. Chin. J. Evid Based. Med. 1 (08), 606–613. 10.3969/j.issn.1672-2531.2007.08.014

[B74] Wu J. C. Zhang X. Wang J. H. Liu Q. W. Wang X. Q. Wu Z. Q. (2019). Gene Polymorphisms and Circulating Levels of the TNF-Alpha Are Associated with Ischemic Stroke: A Meta-Analysis Based on 19,873 Individuals. Int. Immunopharmacol. 75, 105827. 10.1016/j.intimp.2019.105827 31454695

[B75] Wu J. Liu R. Xu D. Li Y. Chang Z. Hao J. (2021). Anti-cerebral Ischemia Mechanisms of Brain Absorption Components of Xingnaojing Injection Based on GC-MS and Network Pharmacology. Chin. Traditional Herbal Drugs 52 (3), 808–820.

[B76] Wu Q. C. (2009). Effect of Xingnaojing Injection on Acute Cerebral Infarction and its Intervention on Inflammatory Factors. J. Emerg. Traditional Chin. Med. 18 (11), 1809–1810. 10.3969/j.issn.1004-745X.2009.11.034

[B77] Wu Q. F. Xu L. F. (2020). Effect of Xingnaojing Injection on Neurological Function Recovery and Anti-inflammatory Effect in Patients with Acute Ischemic Stroke. New J. Tradit. Chin. Med. 52 (9), 52–55. 10.13457/j.cnki.jncm.2020.09.015

[B78] Wu W. Y. Liao J. Huang J. Sun H. Y. (2017). Application and Safety of Xingnao Injection in Patients with Cerebral Infarction. Hebei Med. 23 (8), 1304–1307. 10.3969/j.issn.1006-6233.2017.08.020

[B79] Xiao J. Li F. Cao L. (2018). Effect of Alteplase Combined with Xingnaojing Injection on Inflammatory Reaction and Intracranial Blood Flow Velocity in Patients with Acute Ischemic Stroke. Mod. J. Integrated Traditional Chin. West. Med. 27 (29), 3260–3262. 10.3969/j.issn.1008-8849.2018.29.022

[B80] Xu Y. (2010). Pharmacological and Pharmacodynamic Research and Clinical Application of Xingnaojing Injection. Mod. J. Integrated Traditional Chin. West. Med. 19 (4), 507–510. 10.3969/j.issn.1008-8849.2010.04.095

[B81] Xu Z. Q. Zhou Y. Shao B. Z. Zhang J. J. Liu C. (2019). A Systematic Review of Neuroprotective Efficacy and Safety of DL-3-N-Butylphthalide in Ischemic Stroke. Am. J. Chin. Med. 47 (03), 507–525. 10.1142/S0192415X19500265 30966774

[B82] Yang N. Li X. (2015). Clinical Effect of Xingnaojing Combined with Nimodipine on Patients with Acute Cerebral Infarction. Chin. J. Mod. Drug Appl. 1 (23), 98–99. 10.14164/j.cnki.cn11-5581/r.2015.23.073

[B83] Yin J. Q. Liu J. (2018). Effect of Xingnaojing Injection on Neurological Deficit and Hemorheology in Patients with Acute Cerebral Infarction. Clin. J. Chin. Med. 10 (27), 34–36.

[B84] Yu F. Liu X. Yang Q. Fu Y. Fan D. (2019). In-hospital Recurrence in a Chinese Large Cohort with Acute Ischemic Stroke. Sci. Rep. 9 (1), 14945–14947. 10.1038/s41598-019-51277-8 31628361 PMC6802201

[B85] Zhang C. Liao Y. Liu L. Sun Y. Lin S. Lan J. (2020). A Network Pharmacology Approach to Investigate the Active Compounds and Mechanisms of Musk for Ischemic Stroke. Evid. Based Complement. Alternat Med. 2020, 4063180. 10.1155/2020/4063180 32714405 PMC7354650

[B86] Zhang L. Ai N. (2016). Effect of Xingnaojing Injection on Serum Copeptin, NT proBNP Levels and NIHSS Score in Patients with Acute Cerebral Infarction. Hebei Med. J. 38 (14), 2149–2151. 10.3969/j.issn.1002-7386.2016.14.019

[B87] Zhang X. (2020). Effect of Xingnaojing Injection on Acute Cerebral Infarction. China Pract. Med. 15 (1), 118–119. 10.14163/j.cnki.11-5547/r.2020.01.055

[B88] Zhang X. L. Zhai L. P. Guan Q. B. Du Y. Y. Qian S. X. (2012). Effect of Xingnaojing Injection on Serum VEGF Expression in Patients with Acute Cerebral Infarction. Zhejiang J. Integrated Traditional Chin. West. Med. 1 (11), 846–848. 10.3969/j.issn.1005-4561.2012.11.005

[B89] Zhang Y Y Y. Y. Shen C. Zhang Y. Liu J. P. (2020). Misunderstanding of Taking "total Effective Rate" as the Evaluation index of Curative Effect of Traditional Chinese Medicine. Chin. J. Drug Eval. 37 (5), 337. 10.3969/j.issn.2095-3593.2020.05.005

[B90] Zhang Y. M. Qu X. Y. Tao L. N. Zhai J. H. Gao H. Song Y. Q. (2020). XingNaoJing Injection Ameliorates Cerebral Ischaemia/reperfusion Injury via SIRT1-Mediated Inflammatory Response Inhibition. Pharm. Biol. 58 (1), 16–24. 10.1080/13880209.2019.1698619 31854225 PMC6968491

[B91] Zhang Y. M. Qu X. Y. Zhai J. H. Tao L. N. Gao H. Song Y. Q. (2018). Xingnaojing Injection Protects against Cerebral Ischemia Reperfusion Injury *via* PI3K/Akt-Mediated eNOS Phosphorylation. Evid. Based Complement. Alternat Med. 2018, 2361046. 10.1155/2018/2361046 30158991 PMC6106974

[B92] Zhao Y. C. (2017). Clinical Observation of Xingnaojing Combined with Butylphthalide in the Treatment of Acute Cerebral Infarction. Nei Mongol J. Traditional Chin. Med. 36 (15), 52–53. 10.3969/j.issn.1006-0979.2017.15.045

[B93] Zhao Z. G. (2020). Clinical Observation of Xingnaojing Injection in the Treatment of Acute Cerebral Infarction (Syndrome of Phlegm Heat Combined). Taiyuvan: Shanxi University of traditional Chinese Medicine.

[B94] Zheng Z. X. Zheng C. F. Chen Z. R. (2019). Effect of Xingnaojing Injection on Thrombolysis in Patients with Acute Cerebral Infarction. J. Intern. Intensive Med. 25 (1), 53–55. 10.11768/nkjwzzzz20190117

[B95] Zhong C. Yang J. Xu T. Xu T. Peng Y. Wang A. (2017). Serum Matrix Metalloproteinase-9 Levels and Prognosis of Acute Ischemic Stroke. Neurology 89 (8), 805–812. 10.1212/WNL.0000000000004257 28747453 PMC5580861

