# Supplementary material for: Efficacy and Safety of Xingnaojing Injection for Emergency Treatment of Acute Ischemic Stroke: A Systematic Review and Meta-Analysis
Source: Front Pharmacol. 2022 Mar 24;13:839305. doi: 10.3389/fphar.2022.839305 (PMC8987164; doi:10.3389/fphar.2022.839305)
Supplement: Supplementary file 2 [file DataSheet1.docx]

Supplementary Material

# Supplementary Figures and Tables

## Supplementary Figures


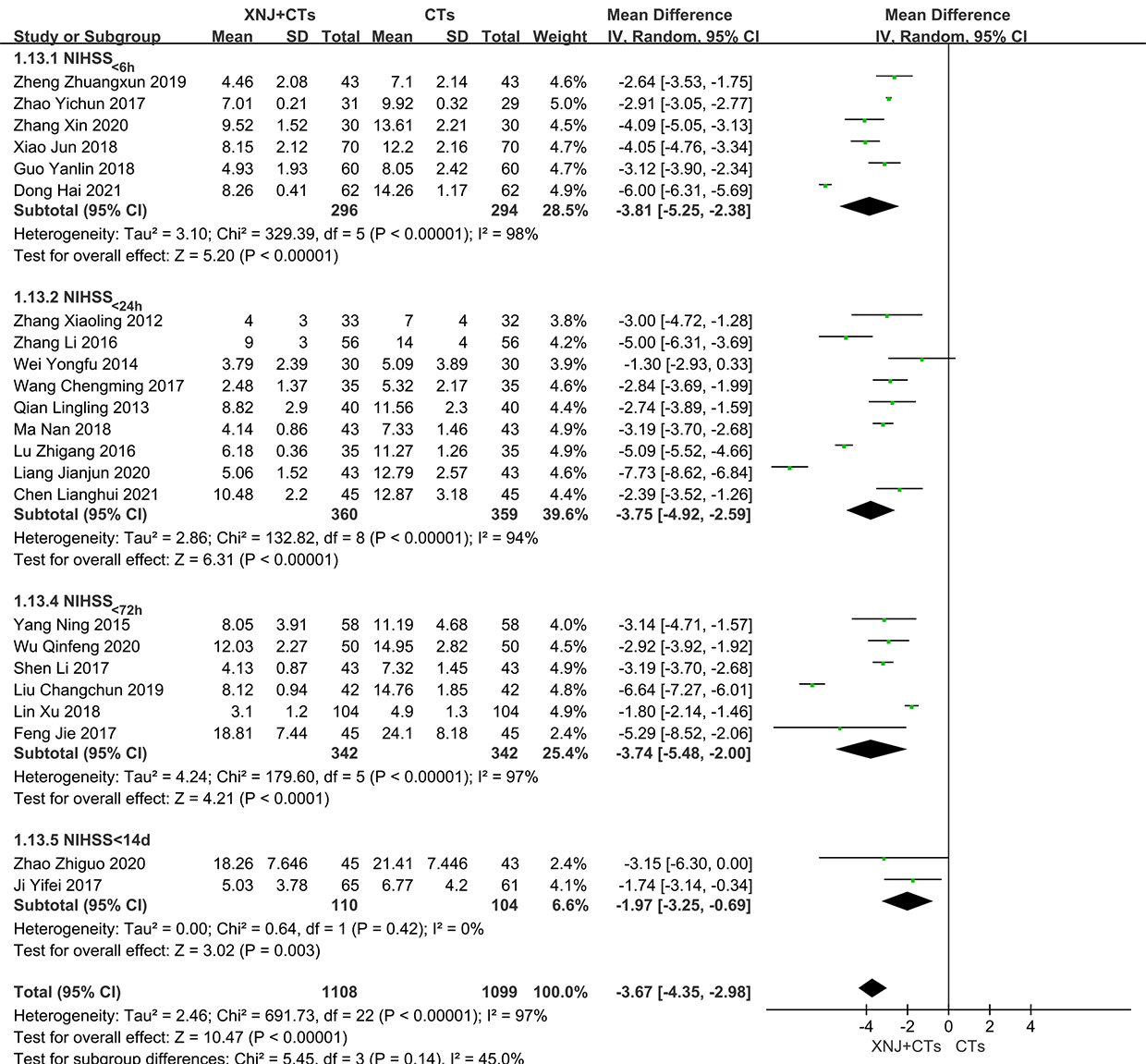


## Supplementary Figure 1. Subgroup analysis of NIHSS at 14 days.

##
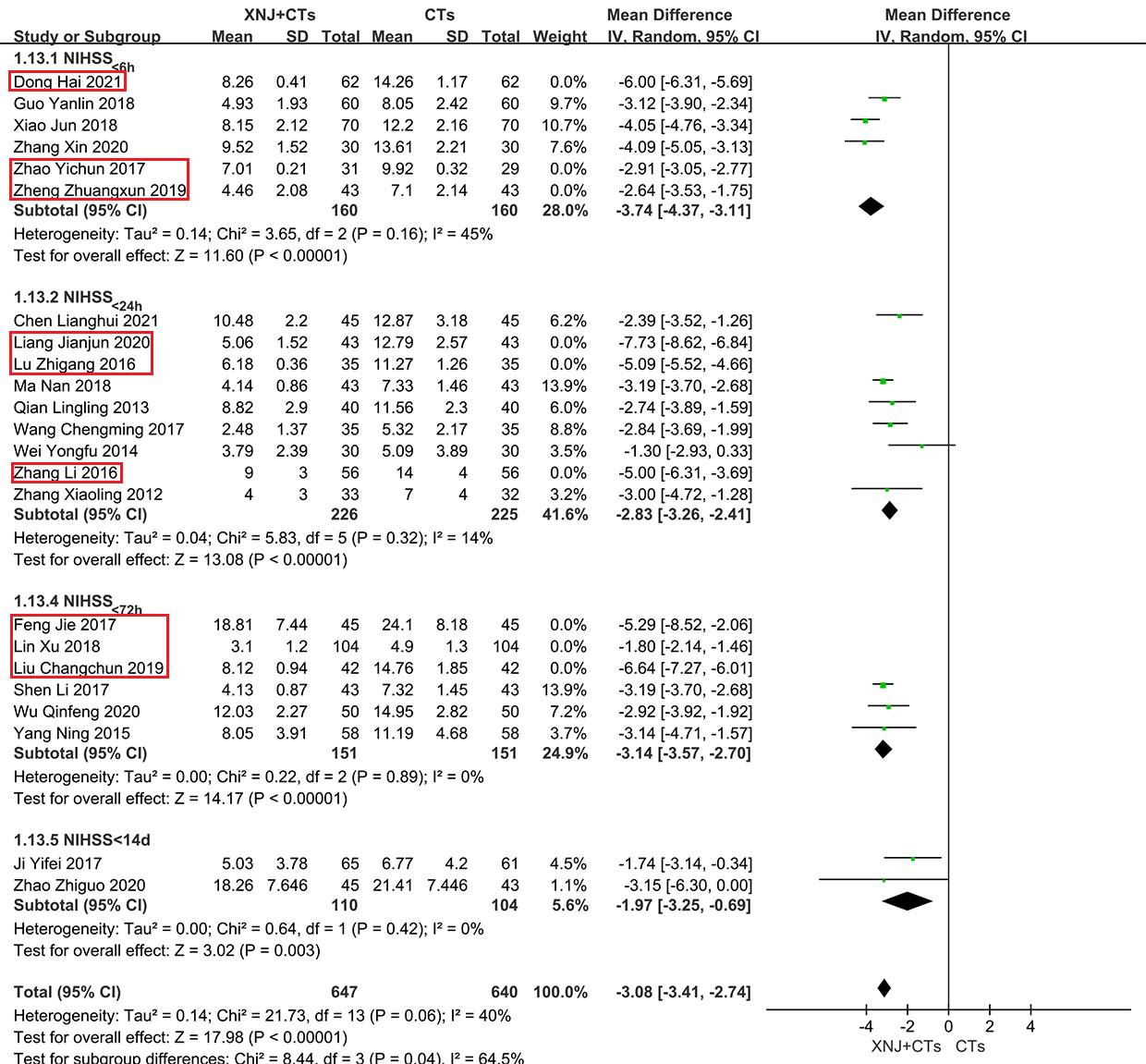
 Supplementary Figure 2. Sensitivity analysis of NIHSS at 14 days.

##
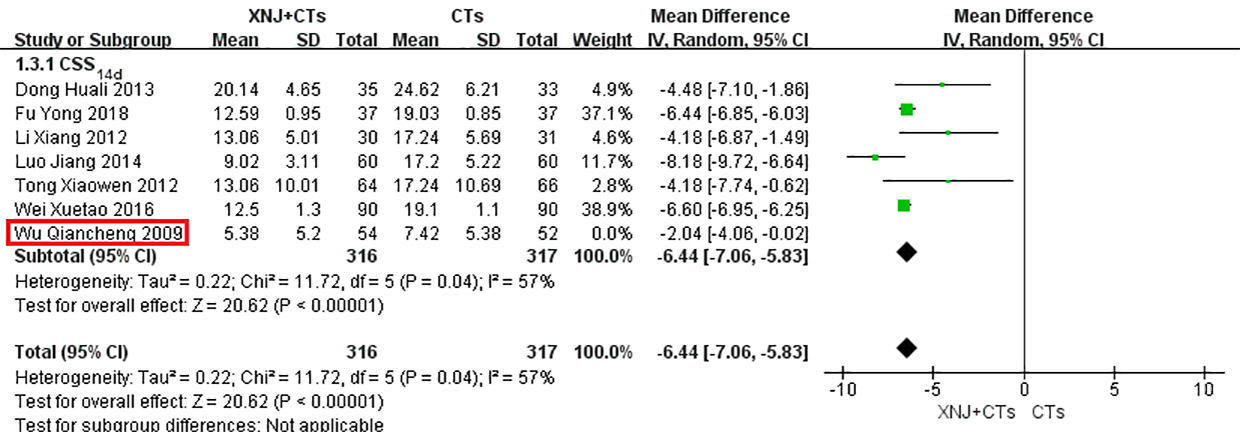


## Supplementary Figure 3. Sensitivity analysis of CSS at 14 days.

##
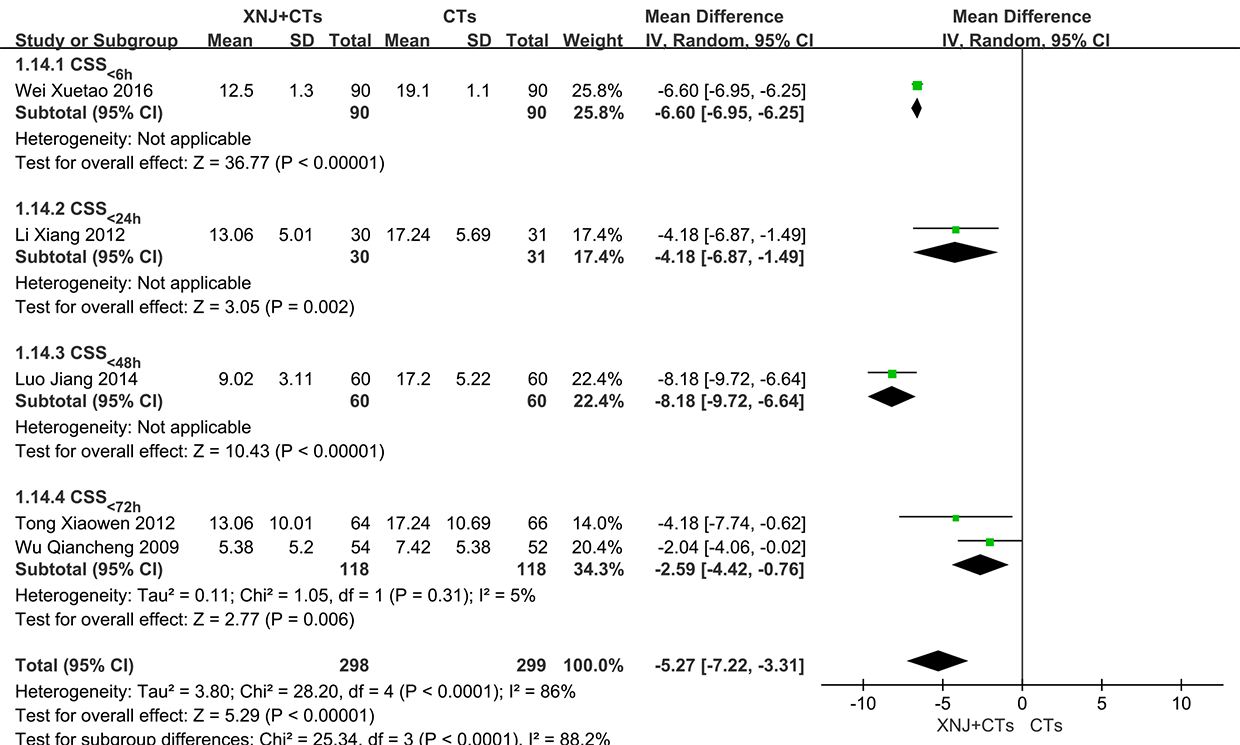


## Supplementary Figure 4. Subgroup analysis of CSS at 14 days.

##
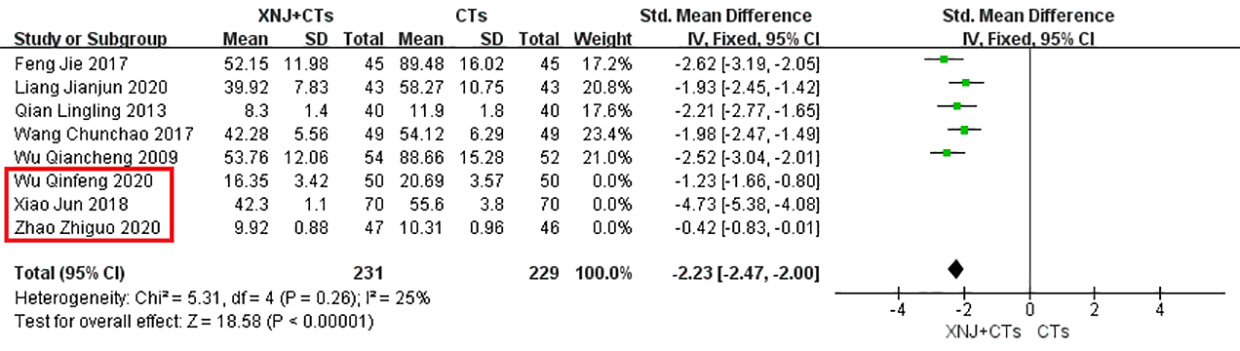


## Supplementary Figure 5. Sensitivity analysis of IL-6.


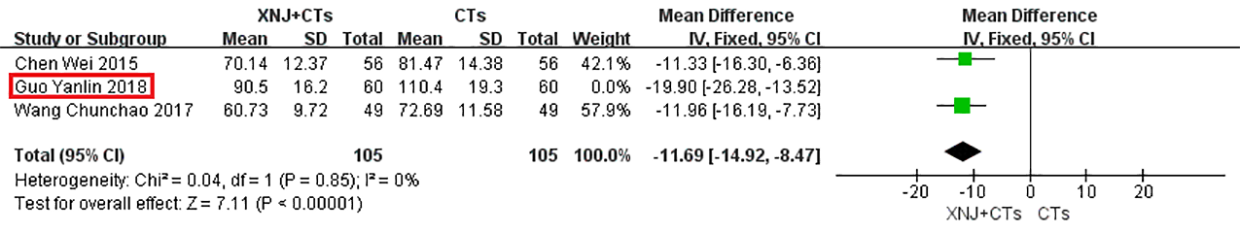


**Supplementary Figure 6.** Sensitivity analysis of MMP-9.

*Note.* Trials highlighted with the red box in Supplementary Figures 2,3,5, and 6, were the removed trials in sensitivity analyses.

## Supplementary Tables

**Supplementary Table 1.** The search strategies for all databases.

| **The search strategy for PubMed** | |
| --- | --- |
| **Number** | **Search terms** |
| #1 | Cerebral infarction[MeSH Terms] |
| #2 | Cerebral infarction[Title/Abstract] |
| #3 | (((((((((((((((((((((((Cerebral Infarctions[Title/Abstract]) OR (Infarctions, Cerebral[Title/Abstract])) OR (Infarction, Cerebral[Title/Abstract])) OR (Cerebral Infarct[Title/Abstract])) OR (Cerebral Infarcts[Title/Abstract])) OR (Infarct, Cerebral[Title/Abstract])) OR (Cerebral Infarction, Left Hemisphere[Title/Abstract])) OR (Left Hemisphere, Infarction, Cerebral[Title/Abstract])) OR (Infarction, Left Hemisphere, Cerebral[Title/Abstract])) OR (Left Hemisphere, Cerebral Infarction[Title/Abstract])) OR (Cerebral, Left Hemisphere, Infarction[Title/Abstract])) OR (Infarction, Cerebral, Left Hemisphere[Title/Abstract])) OR (Subcortical Infarction[Title/Abstract])) OR (Infarction, Subcortical[Title/Abstract])) OR (Infarctions, Subcortical[Title/Abstract])) OR (Subcortical Infarctions[Title/Abstract])) OR (Posterior Choroidal Artery Infarction[Title/Abstract])) OR (Anterior Choroidal Artery Infarction[Title/Abstract])) OR (Cerebral Infarction, Right Hemisphere[Title/Abstract])) OR (Right Hemisphere, Cerebral Infarction[Title/Abstract])) OR (Infarction, Right Hemisphere, Cerebral[Title/Abstract])) OR (Right Hemisphere, Infarction, Cerebral[Title/Abstract])) OR (Cerebral, Right Hemisphere, Infarction[Title/Abstract])) OR (Infarction, Cerebral, Right Hemisphere[Title/Abstract]) |
| #4 | #1 OR #2 OR #3 |
| #5 | stroke[MeSH Terms] |
| #6 | stroke[Title/Abstract] |
| #7 | ((((((((((((((((((((((((((Strokes[Title/Abstract]) OR (Cerebrovascular Accident[Title/Abstract])) OR (CVA (Cerebrovascular Accident)[Title/Abstract])) OR (CVAs (Cerebrovascular Accident)[Title/Abstract])) OR (Cerebrovascular Apoplexy[Title/Abstract])) OR (Apoplexy, Cerebrovascular[Title/Abstract])) OR (Vascular Accident, Brain[Title/Abstract])) OR (Brain Vascular Accident[Title/Abstract])) OR (Brain Vascular Accidents[Title/Abstract])) OR (Brain Vascular Accidents[Title/Abstract])) OR (Cerebrovascular Stroke[Title/Abstract])) OR (Cerebrovascular Strokes[Title/Abstract])) OR (Stroke, Cerebrovascular[Title/Abstract])) OR (Strokes, Cerebrovascular[Title/Abstract])) OR (Apoplexy[Title/Abstract])) OR (Cerebral Stroke[Title/Abstract])) OR (Cerebral Strokes[Title/Abstract])) OR (Stroke, Cerebral[Title/Abstract])) OR (Strokes, Cerebral[Title/Abstract])) OR (Stroke, Acute[Title/Abstract])) OR (Acute Stroke[Title/Abstract])) OR (Acute Strokes[Title/Abstract])) OR (Strokes, Acute[Title/Abstract])) OR (Cerebrovascular Accident, Acute[Title/Abstract])) OR (Acute Cerebrovascular Accident[Title/Abstract])) OR (Acute Cerebrovascular Accidents[Title/Abstract])) OR (Cerebrovascular Accidents, Acute[Title/Abstract]) |
| #8 | #5 OR #6 OR #7 |
| #9 | #4 OR #8 |
| #10 | Xingnaojing[Title/Abstract] |
| #11 | #9 AND #10 |
| **The search strategy for Embase** | |
| **Number** | **Search terms** |
| #1 | 'cerebral infarction'/exp |
| #2 | 'Cerebral Infarctions':ti,ab,kw OR 'Infarctions, Cerebral':ti,ab,kw OR 'Infarction, Cerebral':ti,ab,kw OR 'Cerebral Infarct':ti,ab,kw OR 'Cerebral Infarcts':ti,ab,kw OR ' Infarct, Cerebral':ti,ab,kw OR 'Infarcts, Cerebral':ti,ab,kw OR 'Cerebral Infarction, Left Hemisphere':ti,ab,kw OR 'Left Hemisphere, Infarction, Cerebral':ti,ab,kw OR 'Infarction':ti,ab,kw OR 'Left Hemisphere, Cerebral Left Hemisphere, Cerebral Infarction':ti,ab,kw OR 'Cerebral, Left Hemisphere, Infarction':ti,ab,kw OR 'Infarction, Cerebral, Left Hemisphere':ti,ab,kw OR 'Subcortical Infarction':ti,ab,kw OR 'Infarction, Subcortical':ti,ab,kw OR 'Infarctions, Subcortical':ti,ab,kw OR 'Subcortical Infarctions':ti,ab,kw OR 'Posterior Choroidal Artery Infarction':ti,ab,kw OR 'Anterior Choroidal Artery Infarction':ti,ab,kw OR 'Cerebral Infarction, Right Hemisphere':ti,ab,kw OR 'Right Hemisphere, Cerebral Infarction':ti,ab,kw OR 'Infarction, Right Hemisphere, Cerebral':ti,ab,kw OR 'Right Hemisphere, Infarction, Cerebral':ti,ab,kw OR 'Cerebral, Right Hemisphere, Infarction':ti,ab,kw OR 'Infarction, Cerebral, Right Hemisphere':ti,ab,kw |
| #3 | 'Stroke'/exp |
| #4 | 'Cerebrovascular Accident':ti,ab,kw OR 'Cerebrovascular Accidents':ti,ab,kw OR ' CVA (Cerebrovascular Accident)':ti,ab,kw OR 'CVAs (Cerebrovascular Accident)':ti,ab,kw OR 'Cerebrovascular Apoplexy':ti,ab,kw OR 'Apoplexy, Cerebrovascular':ti,ab,kw OR 'Vascular Accident, Brain':ti,ab,kw OR 'Brain Vascular Accident':ti,ab,kw OR 'Brain Vascular Accidents':ti,ab,kw OR 'Vascular Accidents, Brain':ti,ab,kw OR 'Cerebrovascular Stroke':ti,ab,kw OR 'Cerebrovascular Strokes':ti,ab,kw OR 'Stroke, Cerebrovascular':ti,ab,kw OR 'Strokes, Cerebrovascular':ti,ab,kw OR 'Apoplexy':ti,ab,kw OR 'Cerebral Stroke':ti,ab,kw OR 'Cerebral Strokes':ti,ab,kw OR 'Stroke, Cerebral':ti,ab,kw OR 'Strokes, Cerebral':ti,ab,kw OR 'Stroke, Acute':ti,ab,kw OR 'Acute Stroke':ti,ab,kw OR 'Acute Strokes':ti,ab,kw OR 'Strokes, Acute':ti,ab,kw OR 'Cerebrovascular Accident, Acute':ti,ab,kw OR 'Acute Cerebrovascular Accident':ti,ab,kw OR 'Acute Cerebrovascular Accidents':ti,ab,kw OR 'Cerebrovascular Accidents, Acute':ti,ab,kw |
| #5 | 'Xingnaojing':ti,ab,kw |
| #6 | #1 OR #2 OR #3 OR #4 |
| #7 | #5 AND #6 |
| **The search strategy for Cochrane Library** | |
| **Number** | **Search terms** |
| #1 | MeSH descriptor: [Cerebral Infarction] explode all trees |
| #2 | (Cerebral Infarctions):ti,ab,kw OR (Infarctions, Cerebral):ti,ab,kw OR (Infarction, Cerebral):ti,ab,kw OR (Cerebral Infarct):ti,ab,kw OR (Cerebral Infarcts):ti,ab,kw |
| #3 | (Infarct, Cerebral):ti,ab,kw OR (Infarcts, Cerebral):ti,ab,kw OR (Cerebral Infarction, Left Hemisphere):ti,ab,kw OR (Left Hemisphere, Infarction, Cerebral):ti,ab,kw OR (Infarction, Left Hemisphere, Cerebral):ti,ab,kw |
| #4 | (Left Hemisphere, Cerebral Infarction):ti,ab,kw OR (Cerebral, Left Hemisphere, Infarction):ti,ab,kw OR (Subcortical Infarctions):ti,ab,kw OR (Infarctions, Subcortical):ti,ab,kw OR (Infarction, Subcortical):ti,ab,kw |
| #5 | (Subcortical Infarction):ti,ab,kw OR (Infarction, Cerebral, Left Hemisphere):ti,ab,kw OR (Right Hemisphere, Cerebral Infarction):ti,ab,kw OR (Cerebral Infarction, Right Hemisphere):ti,ab,kw OR (Anterior Choroidal Artery Infarction):ti,ab,kw |
| #6 | (Posterior Choroidal Artery Infarction):ti,ab,kw OR (Infarction, Cerebral, Right Hemisphere):ti,ab,kw OR (Cerebral, Right Hemisphere, Infarction):ti,ab,kw OR (Right Hemisphere, Infarction, Cerebral):ti,ab,kw OR (Infarction, Right Hemisphere, Cerebral):ti,ab,kw |
| #7 | (Xingnaojing):ti,ab,kw |
| #8 | #1 OR #2 OR #3 OR #4 OR #5 OR #6 |
| #9 | #7 AND #8 |
| **The search strategy for CNKI** | |
| (SU = '缺血性脑卒中' OR SU = '缺血性卒中' OR SU = '缺血性脑中风' OR SU = '缺血性中风' OR SU = '缺血性脑血管病' OR SU = '脑梗死' OR SU = '脑梗' OR SU = '脑栓塞' OR SU = '脑缺血' OR SU = '脑卒中' OR SU = '脑梗塞' OR SU = '中风' OR SU = '卒中' OR SU = '小脑梗塞' OR SU = '脑干梗塞' OR SU = '脑中风' OR SU = '脑血管意外' OR SU = '脑血栓') AND (SU= '醒脑静') AND(SU = '急性' OR SU = '超早期' OR SU = '恶化性' OR SU = '早期神经系统功能恶化' OR SU = '超急性期') | |
| **The search strategy for WanFang** | |
| 主题: ("缺血性脑卒中"+"缺血性卒中"+"缺血性脑中风"+"缺血性中风"+"缺血性脑血管病"+"脑梗死"+"脑梗"+"脑栓塞"+"脑缺血"+"脑卒中"+"脑梗塞"+"中风"+"卒中"+"小脑梗塞"+"脑干梗塞"+"脑中风"+"脑血管意外"+"脑血栓") and 主题: ("醒脑静")and 主题：("急性"+"超早期"+"恶化性"+"早期神经系统功能恶化"+"超急性期") | |
| **The search strategy for VIP** | |
| ((M=缺血性脑卒中 OR 缺血性卒中 OR 缺血性脑中风 OR 缺血性中风 OR 缺血性脑血管病 OR 脑梗死 OR 脑梗 OR 脑栓塞 OR 脑缺血 OR 脑卒中 OR 脑梗塞 OR 中风 OR 卒中 OR 小脑梗塞 OR 脑干梗塞 OR 脑中风 OR 脑血管意外 OR 脑血栓) OR (R=缺血性脑卒中 OR 缺血性卒中 OR 缺血性脑中风 OR 缺血性中风 OR 缺血性脑血管病 OR 脑梗死 OR 脑梗 OR 脑栓塞 OR 脑缺血 OR 脑卒中 OR 脑梗塞 OR 中风 OR 卒中 OR 小脑梗塞 OR 脑干梗塞 OR 脑中风 OR 脑血管意外 OR 脑血栓)) AND ((M=醒脑静) OR (R=醒脑静)) | |
| **The search strategy for CBM** | |
| #1 | "缺血性脑卒中"[常用字段:智能] OR "缺血性卒中"[常用字段:智能] OR "缺血性脑中风"[常用字段:智能] OR "缺血性中风"[常用字段:智能] OR "缺血性脑血管病"[常用字段:智能] OR "脑梗死"[常用字段:智能] OR "脑梗"[常用字段:智能] OR "脑栓塞"[常用字段:智能] OR "脑缺血"[常用字段:智能] |
| #2 | "脑卒中"[常用字段:智能] OR "脑梗塞"[常用字段:智能] OR "中风"[常用字段:智能] OR "卒中"[常用字段:智能] OR "小脑梗塞"[常用字段:智能] OR "脑干梗塞"[常用字段:智能] OR "脑中风" |
| #3 | "急性"[常用字段:智能] OR "超早期"[常用字段:智能] OR "恶化性"[常用字段:智能] OR "早期神经系统功能恶化"[常用字段:智能] OR "超急性期"[常用字段:智能] |
| #4 | "醒脑静"[常用字段:智能] |
| #5 | #1 OR #2 |
| #6 | #3 AND #4 AND #5 |

**Supplementary Table 2.** The list of excluded reports.

| **Report excluded** | **Reason** |
| --- | --- |
| Hou Yu 2018 | Unclear outcome |
| Lu Zhigang 2016 | Unclear outcome |
| Cao Jianju 2016 | Unclear outcome |
| Lu Zhigang 2015 | Unclear outcome |
| Lian Jinrong 2010 | Unclear outcome |
| Lu Zhigang 2016 | Duplicate data |
| Tong Xiaowen 2011 | Duplicate data |
| Zhao Jianzhong 2017 | Unavailable data (NIHSS) |
| Xu Guang 2008 | Unavailable data (NIHSS) |
| Yang Xiaomei 2018 | Unavailable data (mRS) |
| Luo Jun 2018 | Unavailable data (mRS) |
| Ma Hongjun 2021 | Incorrect data |
| Li Haijun 2019 | Incorrect interventions (other TCM treatments) |
| Zheng Min 2016 | Incorrect interventions (other TCM treatments) |
| Chen Jieneng 2011 | Incorrect interventions (other TCM treatments) |
| Jiang Changwei 2010 | Incorrect interventions (other TCM treatments) |
| Gu Canli 2009 | Incorrect interventions (other TCM treatments) |
| Gao Fei 2008 | Incorrect interventions (other TCM treatments) |
| Yan Haiqing 2008 | Incorrect interventions (other TCM treatments) |
| Wu Junfeng 2007 | Incorrect interventions (other TCM treatments) |
| Liu Lingling 2018 | Incorrect interventions (unconventional treatments) |
| Li Yunhang 2016 | Incorrect interventions (unconventional treatments) |
| Lou Yifei 2014 | Incorrect interventions (unconventional treatments) |
| Huang Ting 2021 | Incorrect interventions (unconventional treatments) |
| Liu Song 2015 | Incorrect interventions (unclear dosage of Xingnaojing) |
| Yang Chi 2017 | Unclear treatment course |
| Guo Li 2016 | Unclear treatment course |
| Hu Ping 2020 | Less than 10 days of treatment |
| Wu Zuolin 2016 | Unclear participants |
| Li Haibin 2012 | Unclear participants |

**References**

Cao, J. J. (2016). Effect of edaravone, Xingnaojing and clopidogrel on acute cerebral infarction and cerebral nerve function. *Chinese Manipulation and Qi Gong Therapy*. 7(19), 32-32,33

Chen, J. N. (2011). Clinical observation of Xingnaojing Injection in the treatment of 30 cases of acute cerebral infarction. *China Modern Medicine*. 18(7), 76,79. doi: 10.3969/j.issn.1674-4721.2011.07.048

Gu, C. L., Fu, Y. X., Wang, Y. Z., and Wu, J. T. (2009). Effect of Xingnaojing Injection on 85 cases of acute ischemic stroke. *Journal of Emergency in Traditional Chinese Medicine*. 18(12), 1989-1990. doi: 10.3969/j.issn.1004-745X.2009.12.033

Gao, F., Wu, D. F., Xue, Y. D., and Dong, Z. Y. (2008). Effect of Xingnaojing on free radical and clinical efficacy in patients with acute cerebral infarction. *Clinical Medicine*. 28(10), 10-12. doi: 10.3969/j.issn.1003-3548.2008.10.005

Guo, L. (2016). Effect of thrombolytic therapy with alteplase and Xingnaojing in patients with acute cerebral infarction. *Dietary health care*. 3(14), 76-76

Huang, T., Ye, L. Q., and T, Z. (2021). Effects of Xingnaojing Injection on neurological deficit and serum inflammatory mediators in elderly patients with acute cerebral infarction. *Medical Equipment*. 34(13), 95-97.

Hou, Y. (2018). Edaravone injection, Xingnaojing injection combined with clopidogrel tablets in the treatment of 40 cases of acute cerebral infarction. *China Practical Medicine*. 13(9), 94-95. doi: 10.14163/j.cnki.11-5547/r.2018.09.055

Hu, P., and Wan, Y. L. (2020). Effect of Xingnaojing combined with edaravone on serum related factors in patients with acute cerebral infarction. *Clinical Research and Practice*. 5(22), 26-28. doi: 10.19347/j.cnki.2096-1413.202022010

Jiang, C. W., Yang, X. W., Shangguan, Y. P., and Zhao, Y. P. (2010). Clinical effect of Xingnaojing on patients with acute cerebral infarction and recovery of neurological deficit symptoms. *Jiangxi Journal of Traditional Chinese Medicine*. 41(10), 29-30. doi: 10.3969/j.issn.0411-9584.2010.10.016

Lu, Z. G., and Liu, Y. (2016). Neuroprotective effect of Xingnao Kaiqiao method on patients with blood stasis syndrome of acute cerebral infarction and its effect on serum inflammatory factors. *Chinese Archives of Traditional Chinese Medicine*. 34(9), 2178-2180. doi:10.13193/j.issn.1673-7717.2016.09.037

Lu, Z. G., Liu, Y., and Yang, L. X. (2015). Effect of Xingnaojing Injection on cytokines and clinical efficacy in patients with acute cerebral infarction with blood stasis syndrome. *Chinese Journal of Integrated Traditional and Western Medicine in Intensive and Critical Care*. (6), 573-575,576. doi: 10.3969/j.issn.1008-9691.2015.06.004

Lian, J. R., and Dong, H. L. (2010). Clinical observation of Xingnaojing Injection in the treatment of acute cerebral infarction. *Journal of Emergency in Traditional Chinese Medicine*. 19(3), 430-431. doi: 10.3969/j.issn.1004-745X.2010.03.038

Lu, Z. G., Liu, Y., and Lu, Q. (2016). Effect of Xingnaojing Injection on peripheral blood mononuclear cells and T lymphocytes in patients with acute cerebral infarction with blood stasis syndrome. *Chinese Archives of Traditional Chinese Medicine*. 34(12), 3032-3034. doi: 10.13193/j.issn.1673-7717.2016.12.060

Luo, J. (2018). Effect of Xingnaojing Injection on patients with acute ischemic stroke without coma. *Jianghan University*.

Li, H. J., Wang, C. J., and Gao, Z. H. (2019). Effect of Xingnaojing Injection on large area acute cerebral infarction. *World Latest Medicine Information*. 19(38), 157,160. doi: 10.19613/j.cnki.1671-3141.2019.38.109

Liu, L. L. (2018). Effect of Xingnaojing Injection on Hemorheology and neurological function in patients with acute cerebral infarction. *Medical Journal of Chinese People's Health*. 30(24), 101-103. doi: 10.3969/j.issn.1672-0369.2018.24.047

Li, Y. H. (2016). Clinical study on Xingnaojing Injection in the treatment of acute cerebral infarction and control of related risk factors. *Guangxi University of Chinese Medicine*.

Lou, Y. F. (2014). Effect of Xingnaojing Injection on serum IL-1 and IL-6 levels in patients with acute cerebral infarction. *Journal of Emergency in Traditional Chinese Medicine*. 23(5), 984-985. doi: 10.3969/j.issn.1004-745X.2014.05.100

Liu, S. (2015). Effect of Xingnaojing Injection on the levels of hs CRP, IL-6 and TC in patients with acute ischemic stroke. *Acta Neuropharmacologica*. 5(5)

Li, H. B., and Su, X. (2012). Effect of Xingnaojing Injection on serum interleukin-6 and 12 in patients with acute cerebral infarction and its clinical significance. *Capital Medicine* (16), 39-40. doi: 10.3969/j.issn.1005-8257.2012.16.023

Ma, H. J., Sun, H. W., and Li, Y. (2021). Clinical study on Xingnaojing Injection combined with edaravone in treatment of acute cerebral infarction. *Drugs & Clinic*. 36(09), 1922-1926.

Tong, X. W., and Zhu, J. (2011). Effect of Xingnaojing Injection on acute severe cerebral infarction. *Chinese Journal of Integrative Medicine on Cardio-/Cerebrovascuiar Disease*. 09(8), 943-944. doi: 10.3969/j.issn.1672-1349.2011.08.026

Wu, J. F., Zu, H. B., and Chen, Y. H. (2007). Randomized controlled analysis of Xingnaojing in the treatment of 125 cases of severe cerebral infarction. *Nervous Diseases and Mental Health*. 7(6), 442-444. doi: 10.3969/j.issn.1009-6574.2007.06.011

Wu, Z. L., Chen, J., Guo, F. B. and Xu, H. R. (2016). Effect of Xingnaojing on cytokines related to nerve cell repair in elderly patients with cerebral infarction. *Medical Recapitulate*. 22(21), 4312-4315. doi: 10.3969/j.issn.1006-2084.2016.21.042

Xu, G., Chen, J. P., and Xian, Y. (2008). Clinical observation of neuroprotective effect of Xingnaojing Injection on acute ischemic stroke. *Lingnan Journal of Emergency Medicine*. 13(6), 456-457. doi: 10.3969/j.issn.1671-301X.2008.06.026

Yang, X. M. (2018). Study on the treatment of acute ischemic stroke (syndrome of wind phlegm Blocking Collaterals) with Kaiqiao method. *Guangzhou University of Chinese Medicine*.

Yan, H. Q. (2008). Clinical analysis of Xingnaojing in the treatment of 96 cases of cerebral infarction. *Medical Information Section of Operative surgery*. 21(12), 1092-1093. doi: 10.3969/j.issn.1006-1959-C.2008.12.015

Yang, C., and Fu, Y. (2017). Clinical effect of Xingnaojing Injection on acute cerebral infarction. *Chinese Journal of Urban and Rural Enterprise Hygiene*. 32(4), 73-74. doi: 10.16286/j.1003-5052.2017.04.032

Zhao, J. Z. (2017). Clinical observation of Xingnaojing injection combined with brain neurotrophic agent in the treatment of acute cerebral infarction. *Health For Everyone*. (18), 79.

Zheng, M., Ji, X. L., and Wang, H. M. (2016). Clinical study on the effect of Xingnaojing Injection on acute cerebral infarction and its effect on neurological function and biochemical indexes. *Chinese Journal of Cancer Prevention and Treatment*. (S2), 59-60.

**Supplementary Table 3.** The summary table of the studies included.

| **Study** | **Formulation** | **Source** | **Species, concentration** | **Quality control reported?**  **(Y/N)** | **Chemical analysis reported?**  **(Y/N)** | **A chemical characterisation of the preparation** |
| --- | --- | --- | --- | --- | --- | --- |
| He  (2021) | Xingnaojing Injection | Henan Tiandi Pharmaceutical Co., Ltd | - Root of *Curcuma aromatica* Salisb. [Zingiberaceae; Curcumae Radix], 30g - Fruit of *Gardenia jasminoides* J.Ellis [Rubiaceae; Gardeniae Fructus], 30g - Secretion of *Moschus berezovskii* Flerov, *M. sifanicus* Przewalski, or *M. moschiferus* Linnaeus [Cervidae; Moschus], 7.5g - Distillation and recrystallization of leaf of *Dryobalanops aromatica* C.F.Gaertn. [Dipterocarpaceae; Borneolum], 1g | Y – Prepared according to National Drug Standards of China Food and Drug Administration (WS3-B-3353-98-2003) | N | HPLC and GC-MS  1. Curcumae Radix:  curdione, curcumenone, curcumenol, curzerenone and germacrone (anti-inflammation, and improvement of cerebral blood flow);  2. Gardeniae Fructus:  geniposide, eucarvone (antioxidant, anti-inflammation, anticonvulsant);  3. Moschus:  muscone (anti-cerebral ischemia, neuroprotective effects, anti-inflammation, and promoting cell proliferation);  4. Borneolum:  d-borneol, camphor (resistance to reactive oxygen species injury, improvement of cerebral blood flow, inhibition of neuronal excitotoxicity, antagonizing blood-brain barrier injury, and anti-inflammation). |
| Dong et al.  (2021) | Xingnaojing Injection | Wuxi Jimin Kexin Shanhe Pharmaceutical Co., Ltd | - Root of *Curcuma aromatica* Salisb. [Zingiberaceae; Curcumae Radix], 30g - Fruit of *Gardenia jasminoides* J.Ellis [Rubiaceae; Gardeniae Fructus], 30g - Secretion of *Moschus berezovskii* Flerov, *M. sifanicus* Przewalski, or *M. moschiferus* Linnaeus [Cervidae; Moschus], 7.5g - Distillation and recrystallization of leaf of *Dryobalanops aromatica* C.F.Gaertn. [Dipterocarpaceae; Borneolum], 1g | Y – Prepared according to National Drug Standards of China Food and Drug Administration (WS3-B-3353-98-2003) | N |  |
| Chen  (2021) | Xingnaojing Injection | Henan Tiandi Pharmaceutical Co., Ltd | - Root of *Curcuma aromatica* Salisb. [Zingiberaceae; Curcumae Radix], 30g - Fruit of *Gardenia jasminoides* J.Ellis [Rubiaceae; Gardeniae Fructus], 30g - Secretion of *Moschus berezovskii* Flerov, *M. sifanicus* Przewalski, or *M. moschiferus* Linnaeus [Cervidae; Moschus], 7.5g - Distillation and recrystallization of leaf of *Dryobalanops aromatica* C.F.Gaertn. [Dipterocarpaceae; Borneolum], 1g | Y – Prepared according to National Drug Standards of China Food and Drug Administration (WS3-B-3353-98-2003) | N |  |
| Wu and Xu (2020) | Xingnaojing Injection | Wuxi Jimin Kexin Shanhe Pharmaceutical Co., Ltd | - Root of *Curcuma aromatica* Salisb. [Zingiberaceae; Curcumae Radix], 30g - Fruit of *Gardenia jasminoides* J.Ellis [Rubiaceae; Gardeniae Fructus], 30g - Secretion of *Moschus berezovskii* Flerov, *M. sifanicus* Przewalski, or *M. moschiferus* Linnaeus [Cervidae; Moschus], 7.5g - Distillation and recrystallization of leaf of *Dryobalanops aromatica* C.F.Gaertn. [Dipterocarpaceae; Borneolum], 1g | Y – Prepared according to National Drug Standards of China Food and Drug Administration (WS3-B-3353-98-2003) | N |  |
| Zhao  (2020) | Xingnaojing Injection | Wuxi Jimin Kexin Shanhe Pharmaceutical Co., Ltd | - Root of *Curcuma aromatica* Salisb. [Zingiberaceae; Curcumae Radix], 30g - Fruit of *Gardenia jasminoides* J.Ellis [Rubiaceae; Gardeniae Fructus], 30g - Secretion of *Moschus berezovskii* Flerov, *M. sifanicus* Przewalski, or *M. moschiferus* Linnaeus [Cervidae; Moschus], 7.5g - Distillation and recrystallization of leaf of *Dryobalanops aromatica* C.F.Gaertn. [Dipterocarpaceae; Borneolum], 1g | Y – Prepared according to National Drug Standards of China Food and Drug Administration (WS3-B-3353-98-2003) | N |  |
| Zhang (2020) | Xingnaojing Injection | NA | - Root of *Curcuma aromatica* Salisb. [Zingiberaceae; Curcumae Radix], 30g - Fruit of *Gardenia jasminoides* J.Ellis [Rubiaceae; Gardeniae Fructus], 30g - Secretion of *Moschus berezovskii* Flerov, *M. sifanicus* Przewalski, or *M. moschiferus* Linnaeus [Cervidae; Moschus], 7.5g - Distillation and recrystallization of leaf of *Dryobalanops aromatica* C.F.Gaertn. [Dipterocarpaceae; Borneolum], 1g | Y – Prepared according to National Drug Standards of China Food and Drug Administration (WS3-B-3353-98-2003) | N |  |
| Liu  (2020) | Xingnaojing Injection | NA | - Root of *Curcuma aromatica* Salisb. [Zingiberaceae; Curcumae Radix], 30g - Fruit of *Gardenia jasminoides* J.Ellis [Rubiaceae; Gardeniae Fructus], 30g - Secretion of *Moschus berezovskii* Flerov, *M. sifanicus* Przewalski, or *M. moschiferus* Linnaeus [Cervidae; Moschus], 7.5g - Distillation and recrystallization of leaf of *Dryobalanops aromatica* C.F.Gaertn. [Dipterocarpaceae; Borneolum], 1g | Y – Prepared according to National Drug Standards of China Food and Drug Administration (WS3-B-3353-98-2003) | N |  |
| Liang et al.  (2020) | Xingnaojing Injection | Wuxi Jimin Kexin Shanhe Pharmaceutical Co., Ltd | - Root of *Curcuma aromatica* Salisb. [Zingiberaceae; Curcumae Radix], 30g - Fruit of *Gardenia jasminoides* J.Ellis [Rubiaceae; Gardeniae Fructus], 30g - Secretion of *Moschus berezovskii* Flerov, *M. sifanicus* Przewalski, or *M. moschiferus* Linnaeus [Cervidae; Moschus], 7.5g - Distillation and recrystallization of leaf of *Dryobalanops aromatica* C.F.Gaertn. [Dipterocarpaceae; Borneolum], 1g | Y – Prepared according to National Drug Standards of China Food and Drug Administration (WS3-B-3353-98-2003) | N |  |
| Liu et al.  (2019) | Xingnaojing Injection | Henan Tiandi Pharmaceutical Co., Ltd | - Root of *Curcuma aromatica* Salisb. [Zingiberaceae; Curcumae Radix], 30g - Fruit of *Gardenia jasminoides* J.Ellis [Rubiaceae; Gardeniae Fructus], 30g - Secretion of *Moschus berezovskii* Flerov, *M. sifanicus* Przewalski, or *M. moschiferus* Linnaeus [Cervidae; Moschus], 7.5g - Distillation and recrystallization of leaf of *Dryobalanops aromatica* C.F.Gaertn. [Dipterocarpaceae; Borneolum], 1g | Y – Prepared according to National Drug Standards of China Food and Drug Administration (WS3-B-3353-98-2003) | N |  |
| Zheng et al.  (2019) | Xingnaojing Injection | Wuxi Jimin Kexin Shanhe Pharmaceutical Co., Ltd | - Root of *Curcuma aromatica* Salisb. [Zingiberaceae; Curcumae Radix], 30g - Fruit of *Gardenia jasminoides* J.Ellis [Rubiaceae; Gardeniae Fructus], 30g - Secretion of *Moschus berezovskii* Flerov, *M. sifanicus* Przewalski, or *M. moschiferus* Linnaeus [Cervidae; Moschus], 7.5g - Distillation and recrystallization of leaf of *Dryobalanops aromatica* C.F.Gaertn. [Dipterocarpaceae; Borneolum], 1g | Y – Prepared according to National Drug Standards of China Food and Drug Administration (WS3-B-3353-98-2003) | N |  |
| Yin and Liu  (2018) | Xingnaojing Injection | Wuxi Jianhong Pharmaceutical Co., Ltd | - Root of *Curcuma aromatica* Salisb. [Zingiberaceae; Curcumae Radix], 30g - Fruit of *Gardenia jasminoides* J.Ellis [Rubiaceae; Gardeniae Fructus], 30g - Secretion of *Moschus berezovskii* Flerov, *M. sifanicus* Przewalski, or *M. moschiferus* Linnaeus [Cervidae; Moschus], 7.5g - Distillation and recrystallization of leaf of *Dryobalanops aromatica* C.F.Gaertn. [Dipterocarpaceae; Borneolum], 1g | Y – Prepared according to National Drug Standards of China Food and Drug Administration (WS3-B-3353-98-2003) | N |  |
| Ma  (2018) | Xingnaojing Injection | Henan Tiandi Pharmaceutical Co., Ltd | - Root of *Curcuma aromatica* Salisb. [Zingiberaceae; Curcumae Radix], 30g - Fruit of *Gardenia jasminoides* J.Ellis [Rubiaceae; Gardeniae Fructus], 30g - Secretion of *Moschus berezovskii* Flerov, *M. sifanicus* Przewalski, or *M. moschiferus* Linnaeus [Cervidae; Moschus], 7.5g - Distillation and recrystallization of leaf of *Dryobalanops aromatica* C.F.Gaertn. [Dipterocarpaceae; Borneolum], 1g | Y – Prepared according to National Drug Standards of China Food and Drug Administration (WS3-B-3353-98-2003) | N |  |
| Xiao et al.  (2018) | Xingnaojing Injection | Henan Tiandi Pharmaceutical Co., Ltd | - Root of *Curcuma aromatica* Salisb. [Zingiberaceae; Curcumae Radix], 30g - Fruit of *Gardenia jasminoides* J.Ellis [Rubiaceae; Gardeniae Fructus], 30g - Secretion of *Moschus berezovskii* Flerov, *M. sifanicus* Przewalski, or *M. moschiferus* Linnaeus [Cervidae; Moschus], 7.5g - Distillation and recrystallization of leaf of *Dryobalanops aromatica* C.F.Gaertn. [Dipterocarpaceae; Borneolum], 1g | Y – Prepared according to National Drug Standards of China Food and Drug Administration (WS3-B-3353-98-2003) | N |  |
| Guo  (2018) | Xingnaojing Injection | Wuxi Jimin Kexin Shanhe Pharmaceutical Co., Ltd | - Root of *Curcuma aromatica* Salisb. [Zingiberaceae; Curcumae Radix], 30g - Fruit of *Gardenia jasminoides* J.Ellis [Rubiaceae; Gardeniae Fructus], 30g - Secretion of *Moschus berezovskii* Flerov, *M. sifanicus* Przewalski, or *M. moschiferus* Linnaeus [Cervidae; Moschus], 7.5g - Distillation and recrystallization of leaf of *Dryobalanops aromatica* C.F.Gaertn. [Dipterocarpaceae; Borneolum], 1g | Y – Prepared according to National Drug Standards of China Food and Drug Administration (WS3-B-3353-98-2003) | N |  |
| Fu  (2018) | Xingnaojing Injection | Henan Tiandi Pharmaceutical Co., Ltd | - Root of *Curcuma aromatica* Salisb. [Zingiberaceae; Curcumae Radix], 30g - Fruit of *Gardenia jasminoides* J.Ellis [Rubiaceae; Gardeniae Fructus], 30g - Secretion of *Moschus berezovskii* Flerov, *M. sifanicus* Przewalski, or *M. moschiferus* Linnaeus [Cervidae; Moschus], 7.5g - Distillation and recrystallization of leaf of *Dryobalanops aromatica* C.F.Gaertn. [Dipterocarpaceae; Borneolum], 1g | Y – Prepared according to National Drug Standards of China Food and Drug Administration (WS3-B-3353-98-2003) | N |  |
| Lin et al.  (2018) | Xingnaojing Injection | Wuxi Jimin Kexin Shanhe Pharmaceutical Co., Ltd | - Root of *Curcuma aromatica* Salisb. [Zingiberaceae; Curcumae Radix], 30g - Fruit of *Gardenia jasminoides* J.Ellis [Rubiaceae; Gardeniae Fructus], 30g - Secretion of *Moschus berezovskii* Flerov, *M. sifanicus* Przewalski, or *M. moschiferus* Linnaeus [Cervidae; Moschus], 7.5g - Distillation and recrystallization of leaf of *Dryobalanops aromatica* C.F.Gaertn. [Dipterocarpaceae; Borneolum], 1g | Y – Prepared according to National Drug Standards of China Food and Drug Administration (WS3-B-3353-98-2003) | N |  |
| Ji et al.  (2017) | Xingnaojing Injection | Wuxi Jimin Kexin Shanhe Pharmaceutical Co., Ltd | - Root of *Curcuma aromatica* Salisb. [Zingiberaceae; Curcumae Radix], 30g - Fruit of *Gardenia jasminoides* J.Ellis [Rubiaceae; Gardeniae Fructus], 30g - Secretion of *Moschus berezovskii* Flerov, *M. sifanicus* Przewalski, or *M. moschiferus* Linnaeus [Cervidae; Moschus], 7.5g - Distillation and recrystallization of leaf of *Dryobalanops aromatica* C.F.Gaertn. [Dipterocarpaceae; Borneolum], 1g | Y – Prepared according to National Drug Standards of China Food and Drug Administration (WS3-B-3353-98-2003) | N |  |
| Wang et al.  (2017) | Xingnaojing Injection | Wuxi Jimin Kexin Shanhe Pharmaceutical Co., Ltd | - Root of *Curcuma aromatica* Salisb. [Zingiberaceae; Curcumae Radix], 30g - Fruit of *Gardenia jasminoides* J.Ellis [Rubiaceae; Gardeniae Fructus], 30g - Secretion of *Moschus berezovskii* Flerov, *M. sifanicus* Przewalski, or *M. moschiferus* Linnaeus [Cervidae; Moschus], 7.5g - Distillation and recrystallization of leaf of *Dryobalanops aromatica* C.F.Gaertn. [Dipterocarpaceae; Borneolum], 1g | Y – Prepared according to National Drug Standards of China Food and Drug Administration (WS3-B-3353-98-2003) | N |  |
| Wu et al.  (2017) | Xingnaojing Injection | NA | - Root of *Curcuma aromatica* Salisb. [Zingiberaceae; Curcumae Radix], 30g - Fruit of *Gardenia jasminoides* J.Ellis [Rubiaceae; Gardeniae Fructus], 30g - Secretion of *Moschus berezovskii* Flerov, *M. sifanicus* Przewalski, or *M. moschiferus* Linnaeus [Cervidae; Moschus], 7.5g - Distillation and recrystallization of leaf of *Dryobalanops aromatica* C.F.Gaertn. [Dipterocarpaceae; Borneolum], 1g | Y – Prepared according to National Drug Standards of China Food and Drug Administration (WS3-B-3353-98-2003) | N |  |
| Feng et al.  (2017) | Xingnaojing Injection | Henan Tiandi Pharmaceutical Co., Ltd | - Root of *Curcuma aromatica* Salisb. [Zingiberaceae; Curcumae Radix], 30g - Fruit of *Gardenia jasminoides* J.Ellis [Rubiaceae; Gardeniae Fructus], 30g - Secretion of *Moschus berezovskii* Flerov, *M. sifanicus* Przewalski, or *M. moschiferus* Linnaeus [Cervidae; Moschus], 7.5g - Distillation and recrystallization of leaf of *Dryobalanops aromatica* C.F.Gaertn. [Dipterocarpaceae; Borneolum], 1g | Y – Prepared according to National Drug Standards of China Food and Drug Administration (WS3-B-3353-98-2003) | N |  |
| Wang and Lu  (2017) | Xingnaojing Injection | Henan Runhong Pharmaceutical Co., Ltd | - Root of *Curcuma aromatica* Salisb. [Zingiberaceae; Curcumae Radix], 30g - Fruit of *Gardenia jasminoides* J.Ellis [Rubiaceae; Gardeniae Fructus], 30g - Secretion of *Moschus berezovskii* Flerov, *M. sifanicus* Przewalski, or *M. moschiferus* Linnaeus [Cervidae; Moschus], 7.5g - Distillation and recrystallization of leaf of *Dryobalanops aromatica* C.F.Gaertn. [Dipterocarpaceae; Borneolum], 1g | Y – Prepared according to National Drug Standards of China Food and Drug Administration (WS3-B-3353-98-2003) | N |  |
| Zhao  (2017) | Xingnaojing Injection | NA | - Root of *Curcuma aromatica* Salisb. [Zingiberaceae; Curcumae Radix], 30g - Fruit of *Gardenia jasminoides* J.Ellis [Rubiaceae; Gardeniae Fructus], 30g - Secretion of *Moschus berezovskii* Flerov, *M. sifanicus* Przewalski, or *M. moschiferus* Linnaeus [Cervidae; Moschus], 7.5g - Distillation and recrystallization of leaf of *Dryobalanops aromatica* C.F.Gaertn. [Dipterocarpaceae; Borneolum], 1g | Y – Prepared according to National Drug Standards of China Food and Drug Administration (WS3-B-3353-98-2003) | N |  |
| Shen et al.  (2017) | Xingnaojing Injection | Dali Pharmaceutical Co., Ltd | - Root of *Curcuma aromatica* Salisb. [Zingiberaceae; Curcumae Radix], 30g - Fruit of *Gardenia jasminoides* J.Ellis [Rubiaceae; Gardeniae Fructus], 30g - Secretion of *Moschus berezovskii* Flerov, *M. sifanicus* Przewalski, or *M. moschiferus* Linnaeus [Cervidae; Moschus], 7.5g - Distillation and recrystallization of leaf of *Dryobalanops aromatica* C.F.Gaertn. [Dipterocarpaceae; Borneolum], 1g | Y – Prepared according to National Drug Standards of China Food and Drug Administration (WS3-B-3353-98-2003) | N |  |
| Lu et al.  (2016) | Xingnaojing Injection | NA | - Root of *Curcuma aromatica* Salisb. [Zingiberaceae; Curcumae Radix], 30g - Fruit of *Gardenia jasminoides* J.Ellis [Rubiaceae; Gardeniae Fructus], 30g - Secretion of *Moschus berezovskii* Flerov, *M. sifanicus* Przewalski, or *M. moschiferus* Linnaeus [Cervidae; Moschus], 7.5g - Distillation and recrystallization of leaf of *Dryobalanops aromatica* C.F.Gaertn. [Dipterocarpaceae; Borneolum], 1g | Y – Prepared according to National Drug Standards of China Food and Drug Administration (WS3-B-3353-98-2003) | N |  |
| Zhang and Ai  (2016) | Xingnaojing Injection | Wuxi Jimin Kexin Shanhe Pharmaceutical Co., Ltd | - Root of *Curcuma aromatica* Salisb. [Zingiberaceae; Curcumae Radix], 30g - Fruit of *Gardenia jasminoides* J.Ellis [Rubiaceae; Gardeniae Fructus], 30g - Secretion of *Moschus berezovskii* Flerov, *M. sifanicus* Przewalski, or *M. moschiferus* Linnaeus [Cervidae; Moschus], 7.5g - Distillation and recrystallization of leaf of *Dryobalanops aromatica* C.F.Gaertn. [Dipterocarpaceae; Borneolum], 1g | Y – Prepared according to National Drug Standards of China Food and Drug Administration (WS3-B-3353-98-2003) | N |  |
| Wei et al.  (2016) | Xingnaojing Injection | Wuxi Jimin Kexin Shanhe Pharmaceutical Co., Ltd | - Root of *Curcuma aromatica* Salisb. [Zingiberaceae; Curcumae Radix], 30g - Fruit of *Gardenia jasminoides* J.Ellis [Rubiaceae; Gardeniae Fructus], 30g - Secretion of *Moschus berezovskii* Flerov, *M. sifanicus* Przewalski, or *M. moschiferus* Linnaeus [Cervidae; Moschus], 7.5g - Distillation and recrystallization of leaf of *Dryobalanops aromatica* C.F.Gaertn. [Dipterocarpaceae; Borneolum], 1g | Y – Prepared according to National Drug Standards of China Food and Drug Administration (WS3-B-3353-98-2003) | N |  |
| Chen and Wu  (2015) | Xingnaojing Injection | Dali Pharmaceutical Co., Ltd | - Root of *Curcuma aromatica* Salisb. [Zingiberaceae; Curcumae Radix], 30g - Fruit of *Gardenia jasminoides* J.Ellis [Rubiaceae; Gardeniae Fructus], 30g - Secretion of *Moschus berezovskii* Flerov, *M. sifanicus* Przewalski, or *M. moschiferus* Linnaeus [Cervidae; Moschus], 7.5g - Distillation and recrystallization of leaf of *Dryobalanops aromatica* C.F.Gaertn. [Dipterocarpaceae; Borneolum], 1g | Y – Prepared according to National Drug Standards of China Food and Drug Administration (WS3-B-3353-98-2003) | N |  |
| Yang and Li  (2015) | Xingnaojing Injection | NA | - Root of *Curcuma aromatica* Salisb. [Zingiberaceae; Curcumae Radix], 30g - Fruit of *Gardenia jasminoides* J.Ellis [Rubiaceae; Gardeniae Fructus], 30g - Secretion of *Moschus berezovskii* Flerov, *M. sifanicus* Przewalski, or *M. moschiferus* Linnaeus [Cervidae; Moschus], 7.5g - Distillation and recrystallization of leaf of *Dryobalanops aromatica* C.F.Gaertn. [Dipterocarpaceae; Borneolum], 1g | Y – Prepared according to National Drug Standards of China Food and Drug Administration (WS3-B-3353-98-2003) | N |  |
| Luo  (2014) | Xingnaojing Injection | Wuxi Jimin Kexin Shanhe Pharmaceutical Co., Ltd | - Root of *Curcuma aromatica* Salisb. [Zingiberaceae; Curcumae Radix], 30g - Fruit of *Gardenia jasminoides* J.Ellis [Rubiaceae; Gardeniae Fructus], 30g - Secretion of *Moschus berezovskii* Flerov, *M. sifanicus* Przewalski, or *M. moschiferus* Linnaeus [Cervidae; Moschus], 7.5g - Distillation and recrystallization of leaf of *Dryobalanops aromatica* C.F.Gaertn. [Dipterocarpaceae; Borneolum], 1g | Y – Prepared according to National Drug Standards of China Food and Drug Administration (WS3-B-3353-98-2003) | N |  |
| Wei and Cheng  (2014) | Xingnaojing Injection | Wuxi Jimin Kexin Shanhe Pharmaceutical Co., Ltd | - Root of *Curcuma aromatica* Salisb. [Zingiberaceae; Curcumae Radix], 30g - Fruit of *Gardenia jasminoides* J.Ellis [Rubiaceae; Gardeniae Fructus], 30g - Secretion of *Moschus berezovskii* Flerov, *M. sifanicus* Przewalski, or *M. moschiferus* Linnaeus [Cervidae; Moschus], 7.5g - Distillation and recrystallization of leaf of *Dryobalanops aromatica* C.F.Gaertn. [Dipterocarpaceae; Borneolum], 1g | Y – Prepared according to National Drug Standards of China Food and Drug Administration (WS3-B-3353-98-2003) | N |  |
| Qian and Jia  (2013) | Xingnaojing Injection | Wuxi Jimin Kexin Shanhe Pharmaceutical Co., Ltd | - Root of *Curcuma aromatica* Salisb. [Zingiberaceae; Curcumae Radix], 30g - Fruit of *Gardenia jasminoides* J.Ellis [Rubiaceae; Gardeniae Fructus], 30g - Secretion of *Moschus berezovskii* Flerov, *M. sifanicus* Przewalski, or *M. moschiferus* Linnaeus [Cervidae; Moschus], 7.5g - Distillation and recrystallization of leaf of *Dryobalanops aromatica* C.F.Gaertn. [Dipterocarpaceae; Borneolum], 1g | Y – Prepared according to National Drug Standards of China Food and Drug Administration (WS3-B-3353-98-2003) | N |  |
| Dong and Fu  (2013) | Xingnaojing Injection | NA | - Root of *Curcuma aromatica* Salisb. [Zingiberaceae; Curcumae Radix], 30g - Fruit of *Gardenia jasminoides* J.Ellis [Rubiaceae; Gardeniae Fructus], 30g - Secretion of *Moschus berezovskii* Flerov, *M. sifanicus* Przewalski, or *M. moschiferus* Linnaeus [Cervidae; Moschus], 7.5g - Distillation and recrystallization of leaf of *Dryobalanops aromatica* C.F.Gaertn. [Dipterocarpaceae; Borneolum], 1g | Y – Prepared according to National Drug Standards of China Food and Drug Administration (WS3-B-3353-98-2003) | N |  |
| Li et al.  (2012) | Xingnaojing Injection | Wuxi Jimin Kexin Shanhe Pharmaceutical Co., Ltd | - Root of *Curcuma aromatica* Salisb. [Zingiberaceae; Curcumae Radix], 30g - Fruit of *Gardenia jasminoides* J.Ellis [Rubiaceae; Gardeniae Fructus], 30g - Secretion of *Moschus berezovskii* Flerov, *M. sifanicus* Przewalski, or *M. moschiferus* Linnaeus [Cervidae; Moschus], 7.5g - Distillation and recrystallization of leaf of *Dryobalanops aromatica* C.F.Gaertn. [Dipterocarpaceae; Borneolum], 1g | Y – Prepared according to National Drug Standards of China Food and Drug Administration (WS3-B-3353-98-2003) | N |  |
| Zhang et al.  (2012) | Xingnaojing Injection | NA | - Root of *Curcuma aromatica* Salisb. [Zingiberaceae; Curcumae Radix], 30g - Fruit of *Gardenia jasminoides* J.Ellis [Rubiaceae; Gardeniae Fructus], 30g - Secretion of *Moschus berezovskii* Flerov, *M. sifanicus* Przewalski, or *M. moschiferus* Linnaeus [Cervidae; Moschus], 7.5g - Distillation and recrystallization of leaf of *Dryobalanops aromatica* C.F.Gaertn. [Dipterocarpaceae; Borneolum], 1g | Y – Prepared according to National Drug Standards of China Food and Drug Administration (WS3-B-3353-98-2003) | N |  |
| Tong and Zhu  (2012) | Xingnaojing Injection | Wuxi Jimin Kexin Shanhe Pharmaceutical Co., Ltd | - Root of *Curcuma aromatica* Salisb. [Zingiberaceae; Curcumae Radix], 30g - Fruit of *Gardenia jasminoides* J.Ellis [Rubiaceae; Gardeniae Fructus], 30g - Secretion of *Moschus berezovskii* Flerov, *M. sifanicus* Przewalski, or *M. moschiferus* Linnaeus [Cervidae; Moschus], 7.5g - Distillation and recrystallization of leaf of *Dryobalanops aromatica* C.F.Gaertn. [Dipterocarpaceae; Borneolum], 1g | Y – Prepared according to National Drug Standards of China Food and Drug Administration (WS3-B-3353-98-2003) | N |  |
| Chen et al.  (2012) | Xingnaojing Injection | Wuxi Jimin Kexin Shanhe Pharmaceutical Co., Ltd | - Root of *Curcuma aromatica* Salisb. [Zingiberaceae; Curcumae Radix], 30g - Fruit of *Gardenia jasminoides* J.Ellis [Rubiaceae; Gardeniae Fructus], 30g - Secretion of *Moschus berezovskii* Flerov, *M. sifanicus* Przewalski, or *M. moschiferus* Linnaeus [Cervidae; Moschus], 7.5g - Distillation and recrystallization of leaf of *Dryobalanops aromatica* C.F.Gaertn. [Dipterocarpaceae; Borneolum], 1g | Y – Prepared according to National Drug Standards of China Food and Drug Administration (WS3-B-3353-98-2003) | N |  |
| Guan  (2011) | Xingnaojing Injection | NA | - Root of *Curcuma aromatica* Salisb. [Zingiberaceae; Curcumae Radix], 30g - Fruit of *Gardenia jasminoides* J.Ellis [Rubiaceae; Gardeniae Fructus], 30g - Secretion of *Moschus berezovskii* Flerov, *M. sifanicus* Przewalski, or *M. moschiferus* Linnaeus [Cervidae; Moschus], 7.5g - Distillation and recrystallization of leaf of *Dryobalanops aromatica* C.F.Gaertn. [Dipterocarpaceae; Borneolum], 1g | Y – Prepared according to National Drug Standards of China Food and Drug Administration (WS3-B-3353-98-2003) | N |  |
| Wu  (2009) | Xingnaojing Injection | Wuxi Jimin Kexin Shanhe Pharmaceutical Co., Ltd | - Root of *Curcuma aromatica* Salisb. [Zingiberaceae; Curcumae Radix], 30g - Fruit of *Gardenia jasminoides* J.Ellis [Rubiaceae; Gardeniae Fructus], 30g - Secretion of *Moschus berezovskii* Flerov, *M. sifanicus* Przewalski, or *M. moschiferus* Linnaeus [Cervidae; Moschus], 7.5g - Distillation and recrystallization of leaf of *Dryobalanops aromatica* C.F.Gaertn. [Dipterocarpaceae; Borneolum], 1g | Y – Prepared according to National Drug Standards of China Food and Drug Administration (WS3-B-3353-98-2003) | N |  |
